# Implications of power imbalance in antenatal care seeking among pregnant adolescents in rural Tanzania: A qualitative study

**DOI:** 10.1371/journal.pone.0250646

**Published:** 2021-06-30

**Authors:** Wemaeli Mweteni, Julieth Kabirigi, Dismas Matovelo, Rose Laisser, Victoria Yohani, Girles Shabani, Prosper Shayo, Jennifer Brenner, Katie Chaput

**Affiliations:** 1 Department of Community Health, Research and Consultancies, Bugando Medical Centre (BMC), Mwanza, Tanzania; 2 Department of Pediatrics, Catholic University of Health and Allied Sciences (CUHAS), Mwanza, Tanzania; 3 Department of Gynecology and Obstetrics, Catholic University of Health and Allied Sciences (CUHAS), Mwanza, Tanzania; 4 School of Nursing, Catholic University of Health and Allied Sciences (CUHAS), Mwanza, Tanzania; 5 Departments of Pediatrics, and Community Health Sciences, Cumming School of Medicine, University of Calgary, Calgary, Alberta, Canada; 6 Departments of Obstetrics and Gynecology and Community Health Sciences, University of Calgary, Calgary, Canada; Children’s Mercy Hospitals and Clinics Department of Pathology and Laboratory Medicine, UNITED STATES

## Abstract

**Background:**

Adolescent girls (10–19 years) are at increased risk of morbidity and mortality from pregnancy and childbirth complications, compared with older mothers. Low and middle-income countries, including Tanzania, bear the largest proportion of adolescent perinatal deaths. Few adolescent girls in Tanzania access antenatal care at health facilities, the reasons for which are poorly understood.

**Methods:**

We conducted a qualitative thematic analysis study of the experiences of pregnant adolescents with accessing antenatal care in Misungwi district, Tanzania. We recruited 22 pregnant or parenting adolescent girls using purposive sampling, and conducted in-depth interviews (IDIs) about antenatal care experiences. IDI data were triangulated with data from eight focus group discussions (FGDs) involving young fathers and elder men/women, and nine key informant interviews (KIIs) conducted with local health care providers. FGDs, KIIs and IDIs were transcribed verbatim in Swahili. Transcripts were then translated to English and analysed using emergent thematic analysis.

**Results:**

Four main themes emerged: 1) Lack of maternal personal autonomy, 2) Stigma and judgment, 3) Vulnerability to violence and abuse, and 4) Knowledge about antenatal care, and highlighted the complex power imbalance that underlies barriers and facilitators to care access at the individual, family/interpersonal, community, and health-systems levels, faced by pregnant adolescents in rural Tanzania.

**Conclusion:**

Adolescent antenatal care-seeking is compromised by a complex power imbalance that involves financial dependence, lack of choice, lack of personal autonomy in decision making, experiences of social stigma, judgement, violence and abuse. Multi-level interventions are needed to empower adolescent girls, and to address policies and social constructs that may act as barriers, thereby, potentially reducing maternal morbidity and mortality in Tanzania.

## Introduction

Globally, pregnancy and childbirth complications are the leading cause of death among adolescent girls (10–19 years of age) [1]. Recent evidence shows that 18.8% of all pregnancies in Africa, 19.3% of those in Sub-Saharan Africa, and 21.5% in East Africa (including Tanzania) are attributed to adolescent girls [2, 3]. Results from the Tanzania Demographics Health Survey (TDHS) indicate that the percentage of adolescent girls in Tanzania aged 15–19 years who became pregnant increased from 23% to 27% between 2010 and 2016 [3]. Low and middle income countries (LMIC) including Tanzania, have the world’s highest maternal mortality rates for pregnant adolescents at 36 per 100 000 births [4]. Adolescent girls in developing countries are twice as likely to die during childbirth as older mothers, and their infants are at 50% increased risk of neonatal mortality [5].

Improving antenatal care (ANC), delivery care and postnatal care attendance is directly associated with decreasing maternal and infant mortality [6]. The World Health Organization (WHO) currently recommends that pregnant women in LMIC attend at least four ANC visits, with at least one in the first trimester [1]. ANC visits are especially important for reinforcing healthy pregnancy practices, discussing delivery/postnatal care and family planning needs, and to allow for screening and treatment of infection, pregnancy-related, and pre-existing conditions, to reduce risks of maternal and infant morbidity and mortality [1]. In Tanzania the proportion of pregnant women achieving at least four ANC visits has increased from 43% in 2010, to 51% in 2016, with 24% of pregnant women attending ANC in the first trimester [3]. However, in the rural Misungwi district, only 41% of pregnant adolescents attended four or more ANC visits in 2016, and only 13% received ANC prior to 12 weeks gestation [7]. The reasons for ANC attendance below the national average and the specific care-seeking barriers experienced by adolescent girls in Misungwi district are largely unexplored.

During adolescence, rapid physical, psychological, social, and emotional changes occur. Adolescents thus often engage in risk-behaviors including sexual risk-taking which may be one contributor to teen pregnancy [8]. Other factors associated with pregnancy among Tanzanian adolescents include lower education, lower income and rural residence. While social independence and decision making increase during adolescence, most teens remain dependent on parents/guardians for food, shelter, education, clothing and health, which may contribute to a unique set of challenges for them in accessing maternal, neonatal and child health services [5, 9]. This dependence, along with social stigma associated with teen pregnancy can impede pregnant adolescents from accessing resources required to successfully seek and engage in adequate ANC. Globally, pregnant adolescents can have difficulty meeting the responsibilities of pregnancy and parenthood, due to their social and cognitive development, lack of independence, resources, and education, and due to social stigma, and lack of family/social support [10]. These challenges may be amplified in LMIC by extreme poverty, sexism, and social constructs in which adolescents lack power to decide if and when to get married and have children, and how many children to have [11]. Further, adolescent girls and young women in LMIC have less power to make their own health decisions compared with men [12]. Socio-cultural factors such as gendered power imbalances have been reported in Bangladesh as contributors to decreased autonomy in adolescent health-seeking behaviour, but less has reported in the East African context [13].

Given the increased risks for maternal and infant mortality among pregnant adolescents, and their increased risk for attending fewer than the recommended four ANC visits, a more nuanced understanding of the unique challenges faced by pregnant adolescents to accessing ANC particularly in LMIC is needed. Improved understanding of complex barriers and facilitators to ANC attendance among pregnant adolescents can inform future targeted interventions, services and policies that can improve access to adequate ANC, and potentially decrease maternal and infant mortality in this vulnerable group. This study explored experiences of pregnant and parenting adolescents to better understand the barriers and facilitators to accessing ANC in Misungwi district, Mwanza Region, Tanzania.

## Materials and methods

### Study design

We conducted an in-depth qualitative thematic analysis study to explore the lived experiences of pregnant adolescents with accessing ANC in the rural Misungwi district, Tanzania. We used the social—ecological model as a conceptual framework, which categorizes barriers and enablers to seeking and obtaining health care on 4 levels: individual, interpersonal and family, community, and system levels [14]. Our study was nested as a sub-study within a larger longitudinal implementation and evaluation of the Mama na Mtoto (“Mother and Child”) intervention in Tanzania, which aimed to improve the delivery of essential health services to pregnant women, mothers, newborns and children under five; and to improve the health practices and utilization of essential health services by the same target groups [15]. This was conducted through implementation of district-led activities to improve the planning and delivery of high-quality facility-based maternal, newborn and child health (MNCH) services combined with strengthening the demand for these services at the community level while increasing linkages between the community and local health facilities through mobilization of a volunteer community health worker network.

### Setting

We conducted our study between July 2018 and September 2019 in the Misungwi district of Northern Tanzania. Misungwi has a population of approximately 400,000 people, over 90% of whom live in rural areas [16]. The district consists of over 724 small hamlets scattered throughout flatland terrain, where piped water, electricity and sanitation facilities are exceptionally rare [10]. Rural households are typically low-income and highly dependent on subsistence farming, pastoralism, petty trade, and fishing. Misungwi district is served by 49 health facilities (43 dispensaries, 4 health centres, 1 government hospital, and 1 private non-profit hospital).

### Sampling and recruitment

We recruited adolescent girls aged 10–19 years who were pregnant or parenting a child aged less than five years at the time of data collection, from four rural villages; Isesa, Buhunda, Nyamayinza and Kijima, using a maximum variation purposive sampling strategy, wherein we sought to include a variety of maternal ages, martial statuses, and levels of education. We obtained permission from the Misungwi District Medical Officer via the Misungwi District Executive Director to the village government, Village leaders, and through Health facilities administration, to conduct the study.

Before recruiting adolescent girls, we held community engagement meetings with community leaders and community members to introduce the study, provide detailed information about the study, and explain its purpose. Community leaders and volunteer community health workers (CHWs), further engaged with community members to generate awareness and support for the study. Volunteer CHWs, who are seen as peers among villagers, were trained on study aims, protocol, and inclusion and exclusion criteria, and helped to identify potentially eligible adolescent participants. Trained research assistants met with prospective participants multiple times: initial visits involved meeting pregnant and parenting adolescents and their parents/partners at their household to provide detailed information about the study, to build trust and rapport, and to prepare for the interview process. If adolescents indicated interest in participating in the study, the research assistant invited them to engage in an in-depth interview (third visit) (IDI) in their preferred language (Swahili, or Sukuma) and at a location of their choice, to ensure comfort.

Using the same method of community engagement, we also identified women and men who were parents, guardians, and in-laws of an adolescent girl who was pregnant or parenting a child under five at the time of data collection, as well as young husbands (aged less than 25 years), and invited them to participate in focus group discussions (FGDs). Finally, we invited local health care workers (nurses, midwives, physicians) to share their perspectives on the barriers and facilitators faced by pregnant adolescents in accessing ANC, in individual key informant interviews (KIIs).

Participants who could not read or write were asked to select a trusted witness who could translate the information written in the consent form, and were asked to sign the consent with a thumbprint. All participants 18 years of age and above signed a written consent with a witness signing as well. Parents/guardians or husbands (18+) signed consent forms for adolescents less than 18 years of age, and the adolescents signed assent forms. Participation was voluntary and only those who fulfilled consent processes were interviewed. Confidentiality was observed and all information given by the participants was de-identified and assigned a pseudonym, or generic title for data analysis and reporting.

### Data collection

We used a narrative data collection approach, supplemented by in-depth, semi-structured interviews for IDIs, and semi-structured interviews for FGD and KIIs. All data collection guides and questions were pilot tested [17]. Tanzanian members of the authorship team (WM, JK, PS) along with trained local research assistants who were fluent in the local languages, conducted all IDIs, FGDs and KIIs, and rotated through the roles of facilitator and note-taker. For IDIs we asked participants to tell us the story of their pregnancy and followed up with open-ended questions about their pregnancy circumstances and experiences, experiences with accessing ANC, decision-making around ANC, knowledge about ANC, and perceptions of their families, communities, and health workers’ attitudes about adolescent pregnancy. Our open-ended in-depth questions were based on the socio-ecological model and explored experiences on the personal, family, community, health-system and societal levels [18]. All interviews and focus groups were conducted by trained and experienced members of the research team.

We conducted IDIs in the location that was selected as most comfortable by the participant. FGDs were held in schools, churches or local leaders’ offices, based on availability and accessibility for the participants. Interviewers explained the aim of the study and led all participants through the informed consent process before interviews or discussions were started. Interviews and FGDs were conducted and audiotaped in the preferred language of the participant (either Sukuma or Swahili), and field notes were taken. Participants were provided a transport allowance of 2000 Tanzanian shillings (equivalent to approximately 1 United States Dollar (USD)) and health care workers were given 5000 Tanzanian shillings (approximately 2 USD).

### Data analysis

All Sukuma interviews were transcribed verbatim in Swahili (as Sukuma is not commonly written) by research assistants who were fluent in both languages. Quality checks of the translations were conducted, wherein a second bilingual research assistant listened to the audio recording while reading the Swahili transcription, and added to or edited the transcript as needed, to ensure accuracy. A discussion among the research assistants was used to finalize the wording in cases where direct translations were challenging. Swahili recordings were transcribed verbatim in Swahili. All transcripts were then translated from Swahili to English by trained bilingual members of the research team for analysis. We then conducted a second set of quality checks by comparing the English translations to the original language recordings in a group that included native speakers of each language, to ensure that meaning nor content were lost in the translation process.

We imported transcripts into NVIVO^®^ 12 to conduct coding and emergent thematic analysis, using a constant comparison technique [19]. Constant comparison was used to ensure consistency in coding and analysis across multiple coders, and a large number of transcripts. The authors read the transcripts a minimum of twice each to familiarize themselves with and become immersed in the data. All members of the research team jointly coded the first three transcripts and used regular meetings to arrive at consensus on codes, and to create a common codebook. Subsequently we coded the remaining transcripts individually, with continuous sharing for consistency of codes. We grouped codes to form broader themes. An iterative process was employed, when needed, to re-categorize and revisit themes. FGD and KII data were triangulated with data obtained from IDIs. Themes and subthemes were organized according to the socio -ecological model (ref).

### Ethics and consent

This study was approved by the Catholic University of Health and Allied Sciences Research & Ethical Committee (CREC/201/2017), National Institute for Medical Research Lake Zone Institutional Review Board (MR/53/100/493), Mbarara University of Science and Technology Research Ethics Committee (MUREC 1/7), Uganda National Council for Science and Technology (SS 4386), and the University of Calgary Conjoint Health Research Ethics Board (REB17-1741). All informants 18 years of age and above signed a written consent with a witness signing as well. Parents/guardians or husbands (18+) signed consent forms for adolescents less than 18 years of age, and the adolescents signed assent forms. Only those adolescents with fulfilled consent requirements took part in the study.

Consent for publication in peer reviewed journals was obtained from all participants for the use of anonymous quotes. All names appearing in this manuscript are pseudonyms, and do not reflect the true identities of the individual participants.

## Results

We conducted 22 IDIs with adolescent girls, eight focus group discussions (FGDs) three with young husbands, three with mothers (or mothers-in-law) of a pregnant or parenting adolescent, and two with fathers (or fathers-in-law) of pregnant or parenting adolescents from the participants’ communities. We completed nine Key Informant Interviews (KIIs) with local CHWs, doctors, midwives and nurses. All IDI participants chose to have their interviews in their homes. IDIs and KIIs ranged in length between 30 and 60 minutes each and FGDs were 75 to 110 minutes. The demographic characteristics of all study participants are summarized in Table 1.

**Table 1:** Participant characteristics.

	*IDIs*		*FGDs*		*KIIs*	
	Mean	(Range)	Mean	(Range)	Mean	(Range)
***Age in years***						
*1st Pregnancy*	17.5	(15–19)	-	-	-	-
*At study intake*	19.4	(17–21)	40.2	(20–91)	35	(25–54)
	**%**	**(n)**	**%**	**(n)**	**%**	**(n)**
***Sex***						
*Female*	100	(22)	36.2	(21)	42.8	(3)
***Marital Status***						
*Single*	14.3	(3)	0	(0)	0	(0)
*In a relationship*	33.3	(7)	7	(12)	28.6	(2)
*Married*	58.4	(11)	47	(81)	71.4	(5)
*Divorced/widow(ed)*	0	(0)	4	(7)	0	(0)
***Education***						
*None*	4.7	(1)	6.9	(4)	0	(0)
*Primary*	52.4	(11)	77.5	(45)	0	(0)
*Secondary*	42.8	(9)	15.5	(9)	57.1	(4)
*Diploma/university*	0	(0)	0	(0)	42.9	(3)
***Children under 5 yr*.**						
*None*	35	(7)	45.8	(22)	57.1	(4)
*One*	65	(13)	39.6	(19)	28.6	(2)
*Two or more*	0	(0)	14.6	(7)	14.3	(1)

Our IDI participants ranged from 15–19 years of age at the time of their first pregnancies. Half of the adolescents interviewed (48.8%) were either single or in a relationship, but unmarried, compared with the elder parents/in-laws who participated in FGDs, of whom 82% were married. All but one of the pregnant adolescents had completed some level of formal schooling, with 5% having completed primary school, and 43% having partially completed secondary school (Table 1).

### Emergent themes

Four primary themes, some with sub-themes, all tied into the dynamics of power balance emerged from the data. Each theme and respective sub-themes (if applicable) are described below, linked to the socio-ecological model, and followed by illustrative quotes.

#### Theme 1: Lack of maternal personal autonomy

We found that pregnant and parenting adolescent girls lacked the power to support themselves and to make and enact decisions around their own antenatal, delivery and postnatal care. Challenges occurred at the Family level, and impacted both unmarried and married adolescents. The majority of adolescent participants lived with their parents and remained dependent on them or their partners and in-laws to fulfill their basic needs including subsistence, shelter, clothing, medical supplies and transport. Adolescents rarely had access to these resources on their own, nor to their own sources of income. At the interpersonal level, the lack of resources was identified as a greater challenge to accessing ANC among unmarried adolescents.

*Sub-theme 1a) Lack of finances and material resources*.

“*The one who gave me the pregnancy is the one who made things very hard for me… he never covered my expenditures*”

- Shija

“*Transport is one of the obstacles*, *it was better if I could be attending here in our village…It was one thousand and five hundred before*, *but it is currently two thousand one way going*, *and two thousand returning*. *Sometimes the ANC visit date reached*, *and my mom had no money*. *I decided to use a bicycle*.”

- Limi

“*[My partner] sent me some money*. *I bought [maternity] clothes … for me*.”

–Holo

“*The one responsible for the pregnant was bringing the needs and requirements at home*. *Till the day I delivered he was there to support me up to now he is still responsible for me*.”

- Ngeke

Further complicating the personal lack of finances and resources, is a scarcity of finances and resources accessible to those upon whom the adolescent is dependent. The adolescent’s pregnancy thus becomes a burden at the family level.

“*Even those aged 19 years they can’t say who is responsible for the pregnancy*. *We take full responsibility like buying clothes and food for our adolescent girls whose partners are unknown*. *It’s hard for girls to speak out*! *to be open*!”

- Elder father

“*… I faced challenges in supporting her*. *I don’t know who her husband is*. *As mothers those are challenges we face*. *That is why we are suffering*.”

- Elder mother

*Sub-theme 1b) Diminished power for decision making*. Participants described decision making about ANC attendance as a family-level decision, ultimately made by the parents, guardian(s) (sometimes in-laws), or husband, and not by themselves.

“*When I got pregnant*, *I told mom I was pregnant and she asked who was responsible*, *I told her the person*. *She told me to start going to clinic*, *I agreed and started going*.”

- Sayi

“*I must go to Misasi health center… because it is my first pregnancy*. *I told my mother about that and she insisted I must go*. *My mother in law forbids me to go*, *what should I do*?”

- Kamundye

“*My father asked me to start attending ANC services*. *He insisted that I go*, *and so I must go*.”

- Shindye

Other members of the family echoed descriptions of how decisions to seek healthcare are made by others on behalf of pregnant adolescents, further illustrating the family-level barriers.

“*We sit down*, *me and my husband to discuss about taking her to clinic*, *and my husband said he will allow me to take her to clinic*.”

–Elder Mother

*Subtheme 1c) Necessity of partner accompaniment*. Laws and regulations formulated at community level necessitate that pregnant women are accompanied by their partners at the first ANC visit. This proves more difficult to pregnant adolescents as they are often unwed, or do not have partners. Thus, when pregnant adolescents do attempt to access ANC, they are often turned away from clinic when they arrive.

“*They told me I should have taken the letter from my hamlet leader if it was true that my boyfriend ran away*, *so I had to go back to my hamlet leader and took the letter*”

- Kabula

“*Recently*, *when a teenager gets pregnant*, *she has to attend the clinic with her partner*. *As [another participant] said*, *most of these girls return home when they break up with their husbands*, *or it might happen that your daughter gets pregnant at home and never mentions the man responsible for that pregnancy*. *She cannot attend clinic without a partner*, *as health workers deny those who attend their first clinic without a partner*. *This is a problem*.”

- Elder father

#### Theme 2) Stigma and judgment

Adolescents expressed experiences of stigmatization within their communities. Their perceptions centered around feelings of being seen differently from other girls their age, and from how they were seen prior to pregnancy, including perceptions of people talking about them or looking at them differently. The experience of stigmatization leads to a reluctance to be seen in their villages, including attendance at ANC clinics, and contributes to a hesitancy to reveal their pregnancies, hindering access to support that is vital to their ability to initiate care.

“*[the community perception] was bad*, *and some tried to advise me to abort it but I refused*. *… I would go to [traditional birth attendant]’s place*… *because I was worried*. *I was worried to go to the health facility*.”

- Limi

“*friends from school are no longer my friends*, *just villagers… there is a difference that they are still students and I am no longer*”

- Kwezi

“*… there is a difference … because of age*, *the young ones below 20*. *[the community] see[s] it as negative*.”

– Nkwaya

“*I got pregnant when I was at school*, *we closed for break and that was when I discovered that I was pregnant*. *I was not happy at all*. *I knew that I lost education…*

*my parents*, *…[the community]*, *they were very furiously provoking me*.”

- Mwija

Parents, in-laws, and other key informants echoed the stories of stigma faced by pregnant adolescents, and their reluctance to be seen in the community as a result. Additionally, it is noted that the stigma is primarily faced due to the reactions of men (rather than women) in the community:

“*…for these early pregnancies*, *she won’t get advice from any one because she hides her pregnancy a lot*, *for example the daughter I am living with*, *after school she won’t even go out to fetch water*, *if you send her to the market*, *she won’t go*”

- Elder mother-in-law

“*And the girls are very scared to come to the clinic alone*, *so you must take her*, *and come with her every month*. *But when you just tell her to go alone*, *she might not reach the clinic*. *She might be so shy*, *because she is so young to be pregnant*. *So she doesn’t want people to see her*.”

- Elder mother

“*They also do not show up early for ANC services as they fear to expose their pregnancies to the community*, *as it is considered as great shame for an adolescent girl to become pregnant while still living at home*. *This affects them a lot in early ANC service visits*, *as they keep thinking how the community will perceive them for being in such a situation at teenage*.”

- Healthcare provider

“*The problem is with men but not us women (laughing)… you might find that he is provoking you*, *especially because of these adolescents who gets pregnant while at school*, *maybe you should give men some seminars too*”

– Elder mother

At times the stigmatization of pregnant adolescents was blamed on the girls themselves, insinuating that their experiences of stigmatization are self-inflicted:

“*…for that case I can say it’s because of stigmatizing themselves*, *that people might see and laugh at them ask why they are pregnant while still very young*. *But we still encourage them through education*, *but I think the barrier is the self-stigmatization and the fear to be seen and laughed at by everybody out there*.”

- Healthcare provider

*Subtheme 2a) Blame*. Pregnant adolescents bear a burden of blame for the pregnancy at family and community levels. Even in cases where the father of the pregnancy abandoned the mother or was unknown to the family, the blame for the pregnancy and the consequences of the pregnancy fell entirely on the pregnant adolescents. If the pregnancy occurs while still in school, the mother is forced to withdraw, ending her formal education, yet the fathers do not face the same consequence. This blame exacerbates fears of revealing pregnancy, which is necessary for adolescents to do early in pregnancy in order that they may seek adequate supports from partners to initiate and attend ANC. This reduces their perceived power in making care-seeking decisions and enacting health behaviours.

“*They were insulting me…*. *they told me I had sex when I was too young…*

*many people… friends at school*.”

- Shindye

“*[My parents] felt so bad because I had to quit my studies because of pregnancy*.”

– Mwalu

“*[My parents] furiously provoked me … that I lost education*”

–Kulwa

“*Honestly*, *we are not happy with these daughters… once it becomes so*, *we decide to be silent*, *but we are not happy about it at all*.”

– Elder father

#### Theme 3: Vulnerability to neglect and abuse

*Subtheme 3a) Abandonment by the partner*. Many adolescent girls lack support from their partners and stories of the partners leaving them once the pregnancy was revealed were common. Partner absence is a barrier to accessing care on its own, however it is exacerbated by the common local by-laws and practices which limit care to women accompanied by a partner. While this practice increases opportunities for HIV testing, and aims to encourage male engagement in MNCH care, prioritizing care to women with partners in attendance has potential for causing unintended harm to pregnant adolescents who already face numerous barriers to accessing care. Pregnant adolescents are sometimes required to get written permission from local leaders to access antenatal care without their partner, which, in light of the stigma they experience, can not only prove daunting, but contributes to late start of ANC, and may discourage some from accessing facility-based ANC.

“*They started searching for my boyfriend who impregnated me in order to take him to court*. *He decided to run away from the village to secure himself from jail*.”

– Kulwa

“*They told me I should have taken the letter from my hamlet leader if it was true that my boyfriend ran away*, *so I had to go back to my hamlet leader and took the letter*”

- Buyegi

“*Before I was pregnant*, *I was happily living with the one I was pregnant with*. *But when I was four months pregnant*, *then he disappeared and looked aside*. *From there the one who was taking care of me was my mother*.”

- Manungwa

“*Adolescent girls who get pregnant while still at school hide their pregnancy and the man who is responsible for her pregnancy always run away once they know their partners are pregnant*.”

- Elder woman

“*I would like to clarify about these girls aged 19 years and below*, *when she gets pregnant and you ask her about the partner*, *partners are nowhere to be found*!”

- Elder woman

Partner abandonment can also exacerbate the dependence of pregnant adolescents on their families, who must then bear the cost of the pregnancy and care of the infant. Further, the Fathers of adolescent girls sometimes face the legal repercussions when there is no adolescent partner to blame:

“*[My father] is the one helping me all this time*, *he has been there for me in difficult times*, *he buys me all the needs I want*.”

- Minza

**“***A case was drawn by school administration*. *My father was taken to Mbarika police post*, *we had to pay some money for his bond*, *and he was released*. *When he returned home*, *they planned to arrest him once again he asked them it will be much wiser to go arrest the father of the man impregnated me instead*.”

- Sato

“*I struggle alone with her … the one who gave her pregnant is living away from where we live so I wasn’t even able to inform him*. *So*, *I had to take her to clinic by myself*. *I struggle with it*. *I incur expenses to pay for the motorbike to take her to clinic*.”

- Elder father

*Subtheme 3b) Fear and experiences of neglect and abuse*. Participants shared stories of their own fears and experiences of neglect and abuse during pregnancy, in addition to their fears that their partners would also be subject to abuse if identified. The pregnancies of adolescent girls, particularly those who are unwed, are not viewed favorably by the community. As a result, pregnant adolescents spoke often of facing negative consequences and punishments from their families, or others. Fears of experiencing neglect or abuse as retribution for their actions were commonly noted as the underlying reason for adolescents to refrain from accessing ANC, or seeking support that was required to attend ANC clinics. Neglect and abuse often directly interfered in their ability to carry out advice (e.g. dietary) received from ANC providers, and further, verbal abuse experienced at the clinics acted as a deterrent to adolescents to complete the recommended number of ANC visits.

“*During my pregnancy*, *I was getting sick frequently but my mother in law was accusing me of lying about it*. *I sometimes spent the whole day without eating anything*, *but I sometimes had to sneak and go to my home place to eat because it is not far from here*… *They [in-laws] treated me like trash*.”

- Nchama

“*… My mother in law chases me out this place often*. *She orders me to go to work even when am seriously sick*. *The first month of my pregnancy I couldn’t eat for weeks*. *One day she came in and woke me up to go to the farm to cultivate*. *I went to the farm while seriously sick*, *I fell down unconscious*, *you see*?”

- Kija

“*There is a girl at my home who previously went away with a man and started their life*, *but when she conceived that man chased her away*. *Then she decided to come back home*”.

– Nchama

“*…[at clinic] when they are asked who their partners are*, *they don’t have anything to say [laughing]*, *so that is very challenging to them*. *And they are sent back home*, *with very harsh words*, *to* “*go back home with your stomach*!”.”

–Mother of adolescent

Pregnant adolescents expressed fears to reveal the names of fathers of their pregnancies, for fear that they would face abusive consequences, which compounds the impact of the abandonment they experience. The men who leave them often escape consequence. As a result, all consequence and responsibility fall on the young mother, further decreasing her power to enact health behaviours and care seeking. Families and others in the community do not acknowledge that these adolescents are victims of abandonment by the father, rather the pregnant adolescents bear the full weight of the actions of two people, and feel powerless to control the father’s involvement. This community-level factor contributes to the difficulty experienced by adolescents in seeking and obtaining the support required to attend ANC.

“*I think adolescent girls do not mention the ones responsible for their pregnancies because they think they have carried a heavy burden wrongly at a tender age*. *She thinks if she mentions the name to her father*, *she might end up bitterly beaten or end up called a prostitute*, *that’s why they decide not to mention the names*”

–Mother of adolescent

#### Theme 4: Knowledge about ANC

Participant stories revealed that pregnant adolescents generally have a good understanding of the reasons and importance of early ANC visits. Many expressed that they understood the importance of ANC for the health of the pregnancy and the baby, and knew that ANC should be accessed early. While this may be a facilitator for them to attempt to initiate and continue attending ANC, the lack of autonomy described above often impedes their ability to attend ANC, even in situations where they are motivated to do so.

“*They examine blood for HIV test*, *after that an expectant mother undergoes abdomen examination before returning back home… It ensures the fetus’ wellbeing in the womb; you can hear the fetus’ pulse*.”

–Mwija

“*They do weight check-ups*, *as well as they follow up the baby’s growth in the womb*. *I was just given [pills] only once… they were helpful for hemoglobin boost…we were also checked for blood pressure*. *[Others] were advising me to attend ANC services but I wasn’t interested*.

–Dotto

Healthcare workers expressed concerns that lack of understanding at the level of the individual adolescent about the importance of ANC were the primary reason that pregnant adolescents did not seek care. However, this view was incongruent with the experiences of the majority of pregnant adolescents, highlighting a lack of understanding among health care workers about the complexity of the financial, and decision-making barriers to care seeking, faced by adolescents, which transcend the interpersonal and family levels.

“*I can say*, *that [not attending ANC] is attributed by lack of education on the importance of ANC services*. *People differ in understanding things*. *If someone understands the importance for ANC services*, *they will obviously not fail to access the facility for ANC services*.”

- Healthcare provider

While lack of knowledge is assumed by some care providers to contribute to reluctance to seek early ANC at the level of the individual adolescent, it was expressed only as a partial reason by our adolescent participants. When questioned about their knowledge of the importance of early ANC, their responses focused on other perceived barriers:

“*Why should I start accessing ANC services at the first month of my pregnancy*? *I wanted some months to pass by*. *How should I attend all the nine ANC services*?”

- Tabu

“*I was worried… there was a lot of terms to adhere to at the nearby facility*. *First*, *I had to go with my partner*, *then paying three thousand as the first service expense and then one thousand per each ANC visit monthly*.”

- Dotto

In some cases, a lack of understanding about the reasons for specific antenatal treatments/supplements were revealed, but they did not hinder ANC attendance or adherence to treatment.

“*The health care providers didn’t tell me exactly what the pills are meant for*. *Yes*, *I take them*, *but I don’t know what they are for*.”

- Sayi

**Fig 1:**
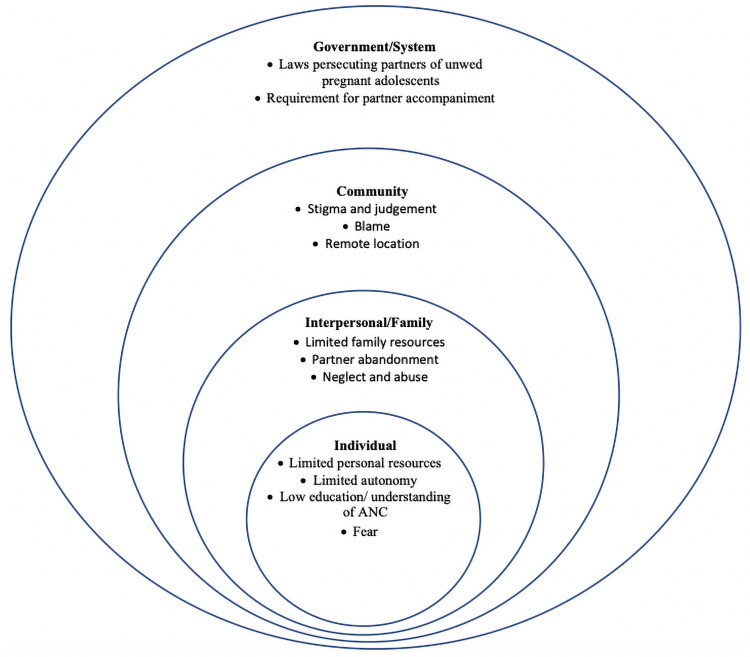
Barriers and enablers categorized within the social ecological model.

A complete list of themes and sub-themes contextualized within the social-ecological model (ref), appears in Fig 1. Our tool captured the data around all the four levels of ecological model. We also recruited participants at all levels, adolescent women at individual level, parents/in-laws and partners at family level, leaders/health workers at system and community level. This model helped to capture the factors around all level to enrich our data as well as confirmability and triangulation of data.

## Discussion

Our study revealed that pregnant adolescent girls in Misungwi, Tanzania face multiple complex challenges to accessing recommended ANC that occur at the individual, interpersonal and family, community, and systems levels. Pregnant adolescents’ stories revealed their dependence on others, including parents and or husbands/partners, their experiences of stigmatization, their vulnerability to neglect and abuse, and a general reluctance to access ANC, based on multiple barriers.

Our thematic analysis results can be synthesized to highlight a complex power imbalance that is faced by pregnant adolescents on multiple levels. The imbalance of power acts to form a network of barriers to accessing ANC. Weber (1947) defines power as "the probability that one actor within a social relationship will be in a position to carry out his or her own will despite resistance, regardless of the basis on which this probability rests " [20]. The things that enable a person to carry out his or her own will, in a given situation, are considered “power sources”, and include knowledge, skills, and physical resources, among others [19]. Thus, the power of any given actor in a relationship can be expressed as a function of the sources of power available to him or her at any given time of need [21]. Power imbalance occurs when power sources are unequally distributed between two or more actors in a given situation [22]. The imbalance of power increases as the availability of power sources to the individual in need decreases [23]. While our study themes can be categorized simply as barriers to accessing ANC for pregnant adolescents occurring at various social levels, an incorporation of the concept of power imbalance in their interpretation allows a much more complete picture of the complexity of the adolescent girl’s pregnancy experience living in Misungwi district to emerge. It also highlights how factors at various social levels interact to limit the power of these pregnant adolescents. This in turn can help to ensure that the findings of this research can inform policy change and interventions that will be effective for optimizing ANC for this population.

Our participants’ experiences and those of their families and community highlight how pregnant adolescents in Misungwi become stuck between childhood dependence and the expectation that they will take full responsibility as mothers. Nearly half of the adolescents in our study became pregnant while still single, and not yet employed. Similar results were reported in a South African study where 94% of pregnant teenage mothers were unemployed and 82.4% were single and remained dependent on others [24]. Financial and material resources are an important power source in the imbalance faced by pregnant adolescents in this setting. Although Tanzania’s National Health System is mandated to provide maternal health services free of charge [25], the reality is that those accessing ANC, delivery and postnatal health care often face considerable financial burden, that leads, in some cases, to families selling crops or other assets or borrowing money to afford the necessary care [26]. The finances necessary to engage in seeking ANC, such as transportation costs, buying maternity clothing, paying for diagnostics and in some cases user-fees, are not directly available to adolescent girls. Thus, the lack of financial independence reduces an adolescent mother’s power to make her own decisions, and take action to access ANC. The power imbalance this causes is exacerbated by partner abandonment, and stigma.

Experiences of stigma were prominent in the stories of our adolescent participants. Pregnancy before marriage, and at a young age remain socially unacceptable. The resulting stigma adolescent girls face, to be seen in their villages, makes it more difficult for them to ask for the assistance and resources they need. A previous qualitative of pregnant 665 adolescents have had the similar findings, that stigma was a significant barrier for unmarried women seeking reproductive healthcare [27]. In addition, partners frequently abandon pregnant adolescents, which not only leaves the young mother to bear the full burden of the stigma, it also decreases the number of people available to her to potentially provide the resources necessary for accessing ANC.

While the assumption was expressed by healthcare workers that pregnant adolescents lack understanding of the importance of ANC, our participants demonstrated very good knowledge about the need for early ANC. This incongruence between the views of healthcare workers and the knowledge level of pregnant adolescents should be addressed, as healthcare workers may otherwise over-invest in trying to educate adolescent girls, which is likely to have little impact on their care seeking, and ANC attendance. Knowledge about adequate ANC as a power source was overshadowed by the adolescent’s lack of decision making ability; participants shared with us how it is a societal norm in Misungwi district for elders (parents, parents-in-law) or husbands to make the decision about whether a pregnant adolescent is allowed to access ANC. Further, some who do seek care are turned away by healthcare staff, and in some cases face abuse as a result of being abandoned by their partners. A previous qualitative studies of pregnant adolescents in have had similar findings, which revealed significant violence and abuse sustained by pregnant adolescents following partner abandonment [28, 29]. It is therefore unlikely that improving education and knowledge about ANC alone, would have much impact on ANC attendance among pregnant adolescents in Misungwi.

Systems-level laws and policies, in place for the general good of the population might also contribute to the power imbalance faced by pregnant adolescents in Tanzania. For example, impregnating or marrying a primary or a secondary school pupil in Tanzania is illegal, and punishable by fines of not less than five million shillings ($2600 US) or a five-year prison term, or both [30]. While this law aims to protect the rights and safety of young girls, it may inadvertently contribute to partners (young or older) of adolescent girls who become pregnant, to abandon them and remain unidentified, and thus uninvolved in the pregnancy. Additionally, in the absence of a male partner, the adolescent girl’s father may be targeted by law enforcement to answer to the legal repercussions of her pregnancy, furthering negative consequences to her family, and potentially exacerbating the stigma and judgement she faces. The fear of these negative repercussions compounds the stigma and shame these adolescent girls face, and may contribute to delay ANC, as they fear revealing the pregnancy early.

The bylaws in many Tanzanian communities requiring husband/partner ANC attendance cause substantial difficulties for the pregnant adolescent population in accessing adequate ANC as nearly half (42%) do not have a partner to accompany them to clinic (Table 1). Single pregnant adolescents attempting to seek care are turned away if their partner is absent, which leads to embarrassment, and undermines personal autonomy.

Thus, partner abandonment, lack of knowledge, resources, and decision-making, as well as fears and experiences of stigma and abuse all act in combination to reduce the power and autonomy of pregnant adolescents with respect to others in the family, the community and the health system. This power imbalance and the resulting barriers to healthcare access may have a substantial impact on the health outcomes of pregnant adolescents and those of their children. Our study themes highlight several barriers to accessing adequate ANC experienced by pregnant adolescents in a rural Tanzanian community occurring at multiple social levels. However, a synthesis of these themes in the context of the social power imbalance experienced by our participants uncovers a more complex picture, which will be necessary to consider if effective interventions and changes are to be made to optimize ANC access for this vulnerable population.

### Strengths and limitations

Although we endeavoured to include the voices of pregnant adolescents aged 10 through 19 in this study, were unable to identify or invite any participants aged 10 to 14 years, which may have narrowed the range of experiences we were able to uncover. Additionally, the authors acknowledge that the conceptualization of pregnancy and childbirth is heavily tied to social and cultural factors which can vary widely between communities. For these reasons, the transferability of our study’s findings beyond rural Tanzania, must be undertaken with caution. However, our results echo those found in qualitative studies of similar topics in other communities in South Africa and Brazil, demonstrating that the transferability of our results is not entirely limited.

Our study used triangulation of data sources, and data collection methods, which adds to the dependability of our results. The use of multiple coders on the team for analysis as well as audit of the coding and analysis by a senior qualitative researcher have ensured the neutrality of our findings. This study also included a broad range of participant characteristics, which has contributed to the breadth and richness of the data.

## Conclusion

Antenatal care access among pregnant adolescents in Misungwi district, Tanzania is mediated by complex personal, family, social and systems-level issues that interact to form complex power imbalances that define the adolescent mother’s pregnancy and antenatal care experiences. The themes that emerged in our study of financial dependence, lack of power in decision making, experiences of stigma and judgement, violence and abuse do compromise access to ANC among adolescent girls. Future interventions and potential bylaw and policy changes are needed to optimize ANC access in this vulnerable population.

Based on our findings, we recommend the development and implementation of interventions and strategies focused on the empowerment of young girls, using an evidence-based framework that targets multiple levels of the social-ecological model, such as Van Eerdewijk et al. (2017) [31].

Individual level strategies should focus on empowering adolescent girls through aiming to normalize pregnancy and ANC, education about reproductive and prenatal health and rewarding ANC attendance. Initiatives that encourage leadership opportunities for young girls may enable them to voice their thoughts and concerns more readily, and improve their personal autonomy and ability to access ANC services. Financial incentives at the personal and family levels could help to alleviate financial and resource barriers that can hinder ANC access.

Community-level education initiatives should focus on creating common community goals to improve child health, and on imparting the knowledge that, while adolescent pregnancy may be undesirable, stigmatizing and shaming pregnant adolescents can harm the mother and unborn child. We recommend a focus on educating elders, and husbands in the community about the harms of social stigma, blame and abuse as well as the importance of access to ANC for the adolescent members of their communities.

At the system level, we acknowledge that the regulations requiring partner attendance at ANC, and the laws persecuting those who impregnate an adolescent were intended to aid in HIV testing and preventing sexual abuse and exploitation of girls, respectively, local and regional authorities and public policy makers should reexamine and restructure regulations, taking into account the unintentional negative impacts they may be having on maternal and infant health. This would aid with reducing the interpersonal barriers of partner abandonment, and family burden indirectly.

## Supporting information

S1 File (DOCX)

## Abbreviations

ANC: Antenatal Care
CHWs: Community Health workers
FGDs: Focused Group Discussions
HCW: Health care workers
IDIs: In-depth Interviews
KIIs: Key informant interviews
