# Supplementary material for: Implications of power imbalance in antenatal care seeking among pregnant adolescents in rural Tanzania: A qualitative study
Source: PLoS One. 2021 Jun 30;16(6):e0250646. doi: 10.1371/journal.pone.0250646 (PMC8244882; doi:10.1371/journal.pone.0250646)
Supplement: S1 File — (DOCX) [file pone.0250646.s001.docx]

**T2_IDI: MWONGOZO WA MAHOJIANO KWA WANAWAKE WA UMRI MDOGO**

| **MUONGOZO WA MAHOJIANO** \|__\|__\|__\|__\| **Vifupisho vya mtafiti** \|__\|__\|__\|  **Vifupisho vya mwandishi** \|__\|__\|__\|  **Kundi la washiriki: (zungusha):** **WASICHANA WADOGO.**  **Audio file**: \|__\|__\|__\|  **Namba ya kijiji:**\|__\|__\| **Tarehe**\|__\|__/__\|__/__\|__\|  **Utangulizi**  Mimi_________________(mwezeshaji) kutoka Chuo kikuu cha Sayansi na tiba Bugando-Mwanza ninafanya kazi na mradi wa Mama na Mtoto.  Mimi __________________________kutoka__________________(mwandishi)  Dhumuni la tafiti hii ni kutathmini namna wasichana wanaopata ujauzito katika umri mdogo wanavyopata changamoto za huduma za afya katika kuhudhuria kliniki wakati wa ujauzito, kujifungua na baada ya kujifungua na nini huwahamasisha. Lengo la mahojiano haya ni kuelewa kiundani changamoto, viwezeshi na vishawishi ambavyo msichana mwenye ujauzito kztikaa umri mdogo hukutana navyo wakati wa kuhudhuria kliniki wakati wa ujauzito, kujifungua na baada ya kujifungua. Mahojiano haya yatachukua saa moja mpaka moja na nusu.   - Mwelekeze mshiriki kujaza taarifa binafsi - M**shiriki ajitambulishe na upitie kiapo cha ridhaa na mshiriki na hakikisha mmejadili vipengele hivi** - **Dhumuni la utafiti** - **Taratibu za utafiti** - **Faida za ushiriki** - **Madhara ya kushiriki** - **Usiri** - **Uhiari wa kushiriki**   Chukua fomu ya ridhaa (moja kwa ajili ya utafiti na nakala moja mpatie mshiriki kama kumbukumbu yake).  Mwelekeze mshiriki kujaza fomu ya taarifa binafsi  (**Maswali ya utangulizi)**  **TAARIFA BINAFSI ZA MSHIRIKI** | |
| --- | --- |
| **Domain** | **Topic and Probes** |
| Uzoefu wa huduma za ujauzito (ANC) | Unaweza kutuambia stori yako ulipogundua kuwa ni mjamzito?   - Ulijisikiaje? - Ulikuwa na umri gani? - Ulimwambia nani na kwa nini? - Je wazazi/mwenzi wako walichukuliaje swala la wewe kuwa mjamzito? - Unahisi jamii inayokuzunguka ilichukuliaje wewe kupata mjamzito? - Unaweza kuniambia kwanini unahisi hivyo kuhusu jamii yako… - Unadhani kuna utofauti jinsi jamii inavyo fikiri juu ya ujauzito kwa wanawake vijana ukilinganisha na wanawake watu wazima? - Ulimuomba nani ushauri? - Ni nani amekuwa mtu wa msaada sana kwako katika kipindi hiki? - Ulipanga kuwa mjamzito?   **Je unafahamu/ulikuwa unafahamu kuhusu upatikanaji wa huduma za afya kipindi cha ujauzito?**  **Dodosa :**   - Je watu wengine walikwambia nini kuhusu huduma za afya kipindi cha ujauzito? - Je, hii ilishawishi vipi mtazamo wako juu ya huduma za afya kipindi cha ujauzito? - **Je ulipanga kuhudhuria kliniki? Kwa nini?** - Je unafikiria huduma za afya kipindi cha ujauzito zina umuhimu gani kwa afya yako? Unaweza kuniambia kwa nini unafikiri hivyo? - Huduma hizi zina umuhimu gani kwa mtoto wako? - Je ni rahisi/si rahisi kwa wewe kwenda kliniki kupata huduma za afya kipindi cha ujauzito? kwanini? - Unahisi ni muhimu kwenda kliniki zote? Na ni mapema kiasi gani unatakiwa kuanza kuhudhuria kliniki wakati wa ujauzito? - Unahisi mwenzi/mpenzi wako anafikiria nini kuhusu kuhudhuria kliniki? Anahusika vipi wakati wa kuhudhuria kliniki? - Unahisi familia yako/ya mwenza wako inafikiria nini kuhusu wewe kuhudhuria kliniki kwa ajili ya huduma za afya kipindi cha ujauzito? - Marafiki zako wanafikiria nini? Je unahisi kuna utofauti kwa wamama watu wazima?kivipi na kwanini? - Je, kuna vitu vyovyote vilisaidia wewe kuhudhuria kliniki kwa ajili ya huduma za afya kipindi cha ujauzito? Kama ndiyo ni vitu gani? Na kivipi? - Je kuna changamoto zozote ulizokutana nazo wakati wa kuhudhuria kliniki? Kwanini/Kivipi? Je ni tofauti kwa kinamama watu wazima? - Unaweza kusema nani alifanya kipindi cha ujauzito wako kuwa kigumu hasa kuhudhuria kliniki? Ni kwa namna gani walisababisha kushindwa kuhudhuria? na kwa nini? |
| Uzoefu wakati wa kujifungua | ***Kama hajawahi kujifungua ana ujauzito wa kwanza uliza mtazamo wake kuhusu kujifungua na huduma baada ya kujifungua (vikwazo, viwezeshi na visababishi)  Unafikiri nini kuhusu kujifungulia kituoni na nyumbani?   - Ungependa kujifungulia wapi kituo cha afya au nyumbani? Kwanini? - Unahisi kuna tofauti kuhusu sehemu ya kujifungulia (kituo cha afya/nyumbani) kwa mama mtu mzima na vile anavyofikiria binti mdogo akiwa mjamzito? - Sehemu ya kujifungulia ni muhimu kiasi gani kwa ajili ya afya ya mwanao? - Sehemu ya kujifungulia ni muhimu kwa kiasi gani kwa ajili ya afya yako? Unaweza kunieleza zaidi?   KWA WENYE UZOEFU WA KUJIFUNGUA  Unaweza kuniambia kuhusu uzoefu wa kujifungua?   - Mwenzi wako alishiriki vipi wakati wa kujifungua?   **Ulizungumza na nani au uliomba ushauri kwa nani kuhusu kujifungua? Walikuambia nini kuhusu kujifungulia kituoni au nyumbani?**   - Je ni rahisi kwa mwanamke wa jamii hii kumweleza mwenzi/mume wake kuwa ameanza kuhisi uchungu wa kujifungua? - Hii huchangia vipi mtazamo wako kuhusu kujifungulia kituoni au nyumbani? - Wanajamii wanafikiria nini kuhusu kujifungulia nyumbani au kituoni? - Unadhani mwenzi au mumeo hufikiria nini kuhusu kujifungulia kituo cha afya au nyumbani? - Je familia yako na familia ya mumeo hufikiri vipi kuhusu kujifungulia kituoni? - Vipi rafiki zako wanafikiri nini kuhusu hilo? Unadhani kuna tofauti ya mawazo yao kati ya mama mtu mzima na binti mdogo? Kivipi? - Unajisikiaje unapomkuta mhudumu wa afya mwanaume/mwanamke? Kwanini?   **Ulijifungulia wapi mtoto wako mdogo (wa mwisho kujifungua)?**   - Unaweza kunielezea ilivyokuwa hadi ukajifungulia nyumbani/njiani/ kituo cha afya?   **Kama alijifungua kituo cha afya**  **(Dodosa):**   - Ni vitu gani vilikuwezesha kujifungulia kituoni? Vilikusaidia kwa namna gani? - Ni nani unafikiri alikua na msaada mkubwa kwako wakati wa kujifungua? Na alikusaidia kwa namna gani? Je ni tofauti kama ingekuwa mama mtu mzima? - Nani alichangia kufanya uzoefu wako wa kujifungua kuwa mgumu? Na kwa namna gani aliyafanya maisha yako kuwa magumu? Je ingekuwa toafauti na ambavyo ingekuwa kwa mama mtu mzima? Kwa nini?   **Kama alijifungulia nyumbani -:**   - Nani unaweza kusema alikuwa wa msaada mkubwa kwako? Ni kwa namna gani alikusaidia? - Ulipitia changamoto yoyote wakati wa kujifungua? - Je, kuna kitu chochote ambacho ni changamoto kilichokufanya usiende kujifungulia kituo cha afya? Kipi na kwanini?) namna gani) (Je, hii ni tofauti na kina mama watu wazima?) - Je, wazazi/mwenza wako alikua na mawazo gani juu ya wewe kujifungulia nyumbani? - Unafikiri watu wa kwenye jamii yako wanafikiriaje kuhusu kujifungulia kwene kituo cha afya/nyumbani? Je, unafikiri hii ni tofauti na ambavyo watu wengine wanafikiri juu ya kujifungulia katika kituo cha afya/nyumbani? (Je, ni tofauti kwa wanawake watu wazima?) - Unafikiri ina umuhimu kiasi gani kwa afya yako kujifungulia kituo cha afya? Unaweza kuniambia ni kwanini unafikiri hivo? - Ina umuhimu gani kwa mtoto wako? - Unafikiri mwenza wako anafikiria nini kuhusu kujifungulia kwenye kituo cha afya? - Alishirikije wakati wa kujifungua? - Unafikiri familia yako/familia ya mwenza wako ilifikiriaje kuhusu wewe kujifungulia kwenye kituo cha afya? - (Marafiki zako wanafikiriaje? "Unafikiri hii ni tofauti kwa wamama watu wazima? Kiaje?) Kwanini? |
| Uzoefu wa huduma za kliniki baada ya kujifungua | *Kama mlengwa ana ujauzito wa kwanza, mulize kuhusu mtazamo wake kuhusu kwenda kliniki baadaa ya kujifungua  Huduma za mama na mtoto baada ya kujifungua ni huduma zote ambazo hutolewa kwa mama na mtoto hadi siku 42 baada ya kujifungua.  Unazionaje huduma za mama na mtoto baada ya kujifungua?  -Unaweza kutueleza kuhusu uzoefu wako kuhusu kliniki ya mama na motto bada ya kujifungua?  -Watu wengine walikuambiaje kuhusu huduma za mama na mtoto baada ya kujifungua?   - Ni kwa namna gani iliathiri mtazamo wako kuhusu huduma za mama na mtoto baada ya kujifungua? - Je ulihitaji kwenda kupata huduma za mama na mtoto baada ya kujifungua? - Je wazazi/walezi/mwenzi wako walikuwa wanawaza nini juu ya wewe kwenda kupata huduma za mama na mtoto baada ya kujifungua? - Unafikiri watu wengine katika jamii wanafikiri nini kuhusu kuhudhuria clini ya huduma za mama na mtoto baada ya kujifungua? Unafikiri kuna utofauti namna wanavyofikiri kuhusu kuhudhuria huduma za mama na mtoto baada ya kujifungua kwa wanawake vijana na wanawake watu wazima? - Nani ulimuomba ushauri kuhusu huduma za mama na mtoto baada ya kujifungua? - Unafikiri ni muhimu kupata huduma za mama na mtoto baada ya kujifungua? Unaweza kuniambia kwa nini umefikiri hivyo? - Ni namna gani ni muhimu kwa mtoto wako? - Unafikiri mwenzi wako anafikiri nini juu ya kuhudhuria kliniki ya huduma za mama na mtoto baada ya kujifungua? Anashirikije? - Ni vitu gani vilikuwa vya msaada kwako katika kupata huduma za mama na mtoto baada ya kujifungua?Ni kwa jinsi gani? Kwa nini umefikiri hivyo? - Nani unayeweza kusema alikuwa wa msaada mkubwa katika kupata huduma za mama na mtoto baada ya kujifungua? Kwa nini? Ni kwa jinsi gani alikusaidia? Je ni tofauti kwa wakina mama watu wazima? - Ni vitu gani vilikuwa vikwazo kwako katika kupata huduma za kwako na mtoto baada ya kujifungua? Ni kwa jinsi gani? Kwa nini unafikiri hivyo? - Nani unaweza kusema alikufanya uone uzoefu wako kuwa mgumu? Na kwa nini? Inaweza kuwa tofauti kama ingekuwa ni mwanamke mtu mzima? |
| Kwa wanawake wenye ujauzito wa kwanza | MTAZAMO KUHUSU KUJIFUNGUA   - Unafikiri vipi kuhusu kujifungua? Kwanini unafikiri hivyo?   Kama anafikiria kujifungua kituo cha afya   - Kwanini umefikia maamuzi haya? - Umejadili kuhusu hili na mtu yeyote? Ni nani? Alikuwa na maoni gani? - Unadhani kuna kitu kinaweza kukufanya ushindwe kujifungulia kituo cha afya? - Ni kitu gani unafikiri unahitaji ili kujifungulia kituo cha afya? - Ni kwa namna gani utakipata? - Unadhani unaweza kupata msaada kufanikisha lengo lako la kujifungulia kituo cha afya? Unaweza kueleza zaidi? - Nani anaweza kuchangia maamuzi yako ? Kivipi? - Unadhani kuna umuhimu wa kujifungulia kituo cha afya? Unaweza kueleza? - Je ni rahisi kwa wanawake wa jamii hii kuwaeleza waume zao pindi wanapohisi uchungu wa kujifungua?   Kama anategemea kujifungulia nyumbani   - Ulifikiaje maamuzi ya kujifungulia kituo cha afya? - Umeawahi kujadili hili na mtu mwingine? Ni nani? Nini mawazo yake? - Familia yako/ mume wako na jamii yako inachukuliaje hili? (uliza mtazami wa mmoja baada ya mwingine) - Wanawake wa jamii hii hujifungulia wapi hasa? - Ni vitu gani unadhani unahitaji ili ujifungulie kituo cha afya? - Kitu gani kinaweza kukuzuia kujifungulia kituo cha afya? - Unadhani kuna umuhimu wowote kwako kujifungulia kituo cha afya? Unaweza kueleza? - Je ni rahisi kwa mwanamke wa jamii hii kumweleza mume/mwenzi wake anapohisi uchungu wa kujifungua?   MTAZAMO KUHUSU KLINIKI BAADA YA KUJIFUNGUA   - Unafikiri nini kuhusu kwenda kliniki baada ya kujifungua?   Dodoso  Je unatarajia kurudi kituo cha afya baada ya kujifungua?  Kama anatarajia kwenda kliniki baada ya kujifungua   - Ulifikiaje maamuzi haya? - Umewahi kujadili na mtu mwingine? Ni nani na alikuwa na mtazamo gani? - Unadhani kuna kitu kinaweza kukufanya ushindwe kwenda kliniki ya mama na mtoto baada ya kujifungua? - Ni vitu gani unahitaji ili uweze kwenda kliniki baada ya kujifungua? - Unafikiri utakipataje kitu hicho/vitu hivyo? - Unadhani utapata msada kukamilisha malengo yako ya kuhudhuria kliniki baada ya kujifungua? - Nani anaweza kuchangia maamuzi yako? Kivipi? - Unadhani kuna umuhimu wa kliniki baada ya kujifungua? - Kwa mwanao? - Kwako mwenyewe   Mwezeshaji aulize zaidi kuhusu yafuatayo:   - Hofu ya mtoto kuugua baada ya chanjo - Unafikiri nini kuhusu afya ya mtoto baada ya kupata huduma za kliniki kama chanjo? - Unadhani atakuwa na afya njema zaidi? Kwanini? - Unapendelea kuhudumiwa na mwanaume au mwanamke kituoni? Kwanini? - Unadhani wasichana wenye umri mdogo kama wewe wanafikiri nini kuhusu mhudumu wa afya?   Kama hatarajii kwenda cliniki baada ya kujifungua   - Ulifikiaje maamuzi hayo? - Uliweza kujadili kuhusu hilo na mtu yeyote? Ni nani? Alikuwa na mtazamo gani? - Mwenzi wako/familia na jamii yako ina mtazamo gani kuhusu hili? - Je wanawake wengine wa jamii hii huhudhuria kliniki baada ya kujifungua? - Ni vitu gani unahitaji ili kuweza kuhudhuria kliniki? - Vitu gani vinaweza kukuzuia kuhudhuria kliniki baada ya kujifungua? - Unadhani kuna umuhimu kuhudhuria kliniki baada ya kujifungua? - Kwa afya ya mwanao - Kwa afya yako mwenyewe   Mwezeshaji aulize zaidi kuhusu yafuatayo   - Hofu ya mtoto kuugua zaidi - Unafikiri nini kuhusu afya ya mtoto baada ya kuhudhuria kliniki baada ya kujifungua? - Unadhani atakuwa na afya zaidi? Kwanini? |
|  |  |
| **Mwisho**  Je kuna kitu kingine chochote unachofikiri ni kikwazo, kiwezeshi au kilichokushawishi kuhusu kupata huduma za afya za ujauzito, kujifungua na baada ya kujifungua   - Toa muhutasari - Mshukuru mshiriki - Toa taarifa zaidi za mawasiliano kwa mshiriki | |

Note

***(Guide the discussion to be around individual level, interpersonal and family, community/society and health system level)***

**Individual-** knowledge about importance of health facility delivery, perceived need, comfortability with health care providers, shyness, mistrust, decision autonomy etc.

**Interpersonal and Family level-** family traditions, family support for home delivery, husband’s knowledge and perceptions, influence of mothers in law and other family members and relatives

**Community and social level-** poverty, religious belief, Traditions and cultural practices, influence of neighbors, community by-laws

**Organizational and health system level-** availability of services, youth friendly clinics, behavior of health care providers, sex of health care provider, quality of health services, distance of health facilities

**Team 2: Adolescent Objective**

**IDI Interview guide**

| **IDNO** \|__\|__\|__\|__\|__\|___\|__\| **Facilitator Initials** \|__\|__\|__\|  **Note-taker Initials** \|__\|__\|__\|  **Group of the informant:** **pregnant adolescent women and mothers**  **Date** \|__\|__/__\|__/__\|__\|  **Community No**:_____________  Participant group: (Circle) Adolescent woman  **Introduction**  I am ______________________________ facilitator from CUHAS/Bugando University, Mwanza, working for Mama na Mtoto. I am _______________________________ from CUHAS/Bugando University, Mwanza, working for Mama na Mtoto. Note-taker for this session. General purpose of the study to explore opinions and perceptions and experiences with pregnant and parenting adolescents in relation to barriers, enablers and influencers for seeking ANC, health facility delivery and PNC services among adolescents here in Misungwi district.  **Participants introduce themselves by first name.**  **Review consent form with participant. Be sure to cover all the following points:**   - General purpose of the study - Study procedures - Benefits of participation - Risks of participation - Confidentiality - Voluntary participation - Questions   **Obtain two signed consent forms before proceeding. One copy is for the participant and one copy is for the research team.**  **Participant’s Personal Information (Demographics) to be filled by participants with consent form.** | |
| --- | --- |
| **Domain** | **Topic and Probes** |
| **Perceptions and experiences with ANC** | **Can you tell us your story about when you found out you were pregnant? Probes:**   - How did you feel? - How old were you? - Who did you tell? Why? - How did your parents or partner react to your pregnancy? - What do you think those in your community think/feel about your pregnancy? Can you tell me why you think so? - Do you think there is a difference in how others think about pregnant adolescents compared to pregnant women who are older than you? - Who did you ask for advice? - Who was the most supportive person in your life during your pregnancy? - Did you plan to get pregnant?   **Were you aware of the availability of health services during pregnancy?**  **Probes:**   - What did other people tell you about health care services during pregnancy? - How did this influence your perception of health care services during pregnancy? - Did you plan to attend ANC services during pregnancy? - Do you think healthcare services during pregnancy are important for your health? Can you tell me why you think this way? - How important are these services for your baby? - Is it easy or hard for you to go to the clinic to get ANC during pregnancy? Why? - How important do you feel it is to go to all ANC visits? - How early should pregnant mothers go to ANC? - What do you think your partner thinks about going to the clinic? - How is/was your partner involved when you go/went to ANC? - What do you think your family/partner's family thinks about you going to the clinic for ANC?" - What do your friends think? Do you think this is different for older mothers? How? Why? - Are there other things that were helpful for you to go to the clinic for ANC during pregnancy? If yes, what are they? How was that helpful? - Who would you say is the most helpful to you with your pregnancy, and getting to the clinic and why?" How is he/she helping you? is this different with older mothers? - Are there any challenges that you have encountered with going for ANC? Why? How? Is this different for older mothers? - Who made your pregnancy experience difficult, especially with getting to the clinic for ANC? How did they make it difficult? Is this different with older mothers? |
| **Experiences with delivery** | *****If the participant is pregnant for the first time, ask about her perception of, rather than experiences with, delivery and PNC. *****  **What do/did you think about home delivery and health facility delivery?**   - Do you want to deliver at the health facility or at home? Why? - Do you think there is difference on how older mothers think about delivering at the health facility/home? - How important is the place of delivery for your baby? - How important do you think place of delivery is for your health? Can you tell me why you think this way?   **THOSE WITH DELIVERY EXPERIENCE**  **Can you tell me your experience with delivery?**   - How was your partner involved during delivery?   **Who did you talk to or ask for advice about your delivery? What did they tell you about health facility delivery/home delivery?**   - Is it easier for women in this community to tell the husbands when they start labour pain? - How did this influence your perception of health facility delivery/home delivery? - What were your parents/partner thoughts about you delivering at the health facility/home? - How did your community think/feel about delivering at the health facility/home? - What do you think your partner thinks about delivering at the health facility/home? - What do you think your family/partner's family think about you going to deliver at a health facility/home? - What do your friends think? Do you think this is different for older mothers? How? - How would you feel when you find a male versus female health care provider? And Why?   **Where did you deliver your last child? Probes:**   - How did you end up delivering at home/on the way/health facility?   *If delivered at the health facility:*   - - What things were most helpful for you to deliver at the health facility? How were they helpful?   - Who would you say was the most helpful to you with your delivery and getting to the facility? Why? How was he/she helping you? Is this different with older mothers?   - Who made your experience difficult with your delivery and getting to the clinic? Why? How did he/she make it difficult? Is this different with older mothers? - *If delivered at home:*   - Who would you say was the most helpful to you with your delivery? Why?   - Did you encounter any challenges with your delivery?   - Is there anything else that stopped you from delivering at a health facility? What? How did it influence your place of delivery? Is this the same for older mothers?   - What do you think your family thought about your home delivery?   - What do you think your community thought about the place of your delivery? Is this different for older mothers?   - Is there importance of delivering to health facility for yourself? Unaweza kunieleza kwanini unafikiria hivyo?   - How is it important is facility delivery for your baby?   - What is your spouse’s/ husband thought about facility delivery?   - What would your family think about this?   - What is your friend’s opinion? Would they think differently with elder women? How? |
| **Experience with PNC** | *****If the participant is pregnant for the first time, ask about her perception of, rather than experiences with, delivery and PNC. *****  Post-natal care means services delivered to a woman and the baby up to 42 days after delivery (immunization, growth monitoring, family planning and breast feeding).  **-Would you explain your experience with PNC?**  **-What did other people tell you about PNC services for mothers and for babies?**   - How did this influence your perception about PNC services for mothers and babies? - Did you think you needed to attend PNC? - What did your parents/caregiver/partner think about you attending PNC? - How do you feel those in your community think/feel about attending PNC clinic? Do you think their opinion is different if the mother is older than you? - Who did you ask for advice about PNC for you and you baby? - How important do you think it is for your health to attend PNC clinic? Can you tell me why you think this way? - How important is it for your baby? - What do you think your partner thinks about you attending PNC? How is he involved with PNC services? - Are there things that were helpful for you to be able to attend PNC? if yes, what were they? How were they helpful? - Who would you say was the most helpful to you with your PNC attendance? why? How was he/she helping you? Is this different with older mothers? - Is there anything that makes it more challenging to attend PNC? Why? or How? Is this different from older mothers? - Who would you say made your PNC experience difficult and why? How did he/she make it difficult? Is this different with older mothers? |
| **For women with first pregnancy with no delivery and PNC experience** | **DELIVERY PERCEPTION**   - **What do you think about delivery?**   **Probes**   - **Where do you expect to deliver, Why do you think so?**   **If expect to delivery health Facility**   - How did you come up with this expectation? - Have you discussed this with anybody? Who is that? What is his/her perception? - Is there anything that you think will make it difficult to deliver at Health facility? - What do you think you need for you to deliver at health facility? - How would you get that? - Do you think you will have support to achieve your expectation? From whom? How? - Who could influence your decision? How? - Do you think there is any importance of delivering at health facility? Can you explain? - Is it easier for women in this community to tell the husbands when they start labour pain? How is that? Why?   **If expect to deliver at home**   - How did you come up with this expectation? - Have you discussed this with anybody? Who is that? What is his/her perception? - What does your partner/family/community members think about this? - Where do other women in this community delivery mostly? - What do you think you need to help you deliver at health facility? - What would hinder you to deliver at Health Facility? - Do you think there is any importance of delivering at health facility? Can you explain? - Is it easier for women in this community to tell the husbands when they start labour pain? |
|  | **PNC PERCEPTION**   - **What do you think about going back to health facility after delivery?**   **Probes**   - **Do you plan to go to the health facility after delivery? Why do you think so?**   **If expect to attend PNC**   - How did you come up with this expectation? - Have you discussed this with anybody? Who is that? What is his/her perception? - Is there anything that you think will make it difficult to attend PNC? - What do you think you need for you to attend PNC? - How would you get that? - Do you think you will have support to achieve your expectation? From whom? How? - Who could influence your decision? How? - Do you think there is any importance for attending PNC? Can you explain? – Facilitator ask importance for;   - baby and  - mother.  **Facilitator should probe the following themes:**   - Fear of children becoming sicker following PNC? - What do you think about health of the baby after receiving PNC services i.e.vaccine? - Do you think they will be healthier? If Yes/no Why? - Do you prefer female or male health care provider? Why? - What do you think about other adolescent’s preference about health care provide?   **If does not expect to attend PNC**   - How did you come up with this expectation? - Have you discussed this with anybody? Who is that? What is his/her perception? - What does your partner/family/community members think about this? - Do other women in this community attend PNC? - What do you think you need to help you to attend PNC? - What would hinder you to attend PNC? - Do you think there is any importance for attending PNC? Can you explain? – Facilitator ask importance for;   -baby and  - mother  **Facilitator should probe the following themes:**   - Fear of children becoming sicker following PNC? - What do you think about health of the baby after receiving PNC services? - Do you think they will be healthier? If Yes/no Why? |
| **Closing**  Is there anything else you think is important about barriers and enablers for seeking antenatal care, health facility delivery or post-natal care that we have not talked about?   - Summarise - Thank participant - Provide extra information and contacts to participants | |

**MUONGOZO WA HADITHI ZA UJAUZITO, KUJIFUNGUA NA BAADA YA KUJIFUNGUA**

**IDNO** |__|__|__|__| **Vifupisho vya mtafiti** |__|__|__|

**Vifupisho vya mwandishi** |__|__|__|

**Kundi la washiriki:** **WASICHANA WADOGO.**

**Audio file**: |__|__|__|

**Namba ya kijiji:**|__|__| **Tarehe**|__|__/__|__/__|__|

**Utangulizi**

Mimi_________________(mwezeshaji) kutoka Chuo kikuu cha sayansi na tiba Bugando-Mwanza ninafanya kazi na mradi wa Mama na Mtoto.

Mimi __________________________kutoka__________________(mwandishi)

Dhumuni la tafiti hii ni kutathmini namna wasichana wanaopata ujauzito katika umri mdogo wanavyopata changamoto za huduma za afya katika kuhudhuria kliniki wakati wa ujauzito, kujifungua na baada ya kujifungua na nini huwahamasisha. Lengo la mahojiano haya ni kuelewa kiundani changamoto, viwezeshi na vishawishi ambavyo msichana mwenye ujauzito katika umri mdogo hukutana navyo wakati wa kuhudhuria kliniki wakati wa ujauzito, kujifungua na baada ya kujifungua. Hii ni pamoja na kujua vitu au watu waliokuwa wa msaada kwako au kuhusika katika kufanya maamuzi ili kupata huduma hizo.

- Mshiriki ajitambulishe na upitie kiapo cha ridhaa na mshiriki na hakikisha mmejadili vipengele hivi;
- Dhumuni la utafiti
- Taratibu za utafiti
- Faida za ushiriki
- Madhara ya kushiriki
- Usiri
- Uhiari wa kushiriki

Chukua fomu ya ridhaa (moja kwa ajili ya utafiti na nakala moja mpatie mshiriki kama kumbukumbu yake).

- Mwelekeze mshiriki kujaza fomu ya taarifa binafsi

(**Maswali ya utangulizi)**

- Unapenda nini kwa rafiki yako wa karibu?
- Nani ni mtu wa mfano kwako?

TAARIFA ZA BINAFSI ZA MSHIRIKI

Muelekeze mshiriki kujaza fomu ya taarifa binafsi.

**MUONGOZO WA KUTOA HISTORIA**

Naomba unielezee historia/hadithi yako ya uzazi tangu ulipopata ujauzito, wakati wa kujifungua na baada ya kujifungua (uzooefu wako na mambo yote uliyokutana nayo).

- Naomba unielezee mambo yote yaliyokuwa msaada kwako wakati wa ujauzito, Wakati wa kujifungua, Wakati baada ya kujifungua.
- Naomba unielezee mambo yote yaliyokuwa vikwazo au changamoto kwako wakati wa ujauziito, wakati wa kujifungua na wakati baada ya kujifungua.
- Naomba unielezee ni nani aliyechangia katika kufanya maamuzi yako wakati wa ujauzito, wakati wa kujifungua na wakati baada ya kujifungua.

**Narrative tool Guide to adolescent aged 15-19 years of age.**

Can you tell me about your story since you found out you were pregnant, during pregnancy, delivery and after delivery?

- Can you talk about all the things that helped in any way during the time of pregnancy, delivery and after delivery?
- Can you talk about all the things that were difficult to you during the time of pregnancy, delivery and after delivery?
- Can you tell us about people who contributed to decisions you made all through the time of pregnancy, during delivery and after?

**MUONGOZO WA MAHOJIANO NA WATUMISHI WA AFYA (KII)**

**IDNO** |__|__|__|__| **Vifupisho vya mtafiti** |__|__|__|

**Vifupisho vya mwandishi** |__|__|__|

**Kundi la Mshiriki: (zungusha):** **WATOA HUDUMA ZA AFYA.**

**Audio file**: |__|__|__|

**Namba ya kijiji:**|__|__| **Tarehe**|__|__/__|__/__|__|

**Utangulizi**

Mimi_________________(mwezeshaji) kutoka Chuo kikuu cha sayansi na tiba Bugando-Mwanza ninafanya kazi na mradi wa Mama na Mtoto.

Mimi __________________________kutoka__________________(mwandishi)

Dhumuni la tafiti hii ni kutathmini namna wasichana wanaopata ujauzito katika umri mdogo wanavyopata changamoto za huduma za afya katika kuhudhuria kliniki wakati wa ujauzito, kujifungua na baada ya kujifungua na nini huwahamasisha. Lengo la mahojiano haya ni kuelewa kiundani changamoto, viwezeshi na vishawishi ambavyo msichana mwenye ujauzito kztikaa umri mdogo hukutana navyo wakati wa kuhudhuria kliniki wakati wa ujauzito, kujifungua na baada ya kujifungua. Mahojiano haya yatachukua saa moja mpaka moja na nusu.

- M**shiriki ajitambulishe na upitie kiapo cha ridhaa na mshiriki na hakikisha mmejadili vipengele hivi**
- **Dhumuni la utafiti**
- **Taratibu za utafiti**
- **Faida za ushiriki**
- **Madhara ya kushiriki**
- **Usiri**
- **Uhiari wa kushiriki**

Chukua fomu ya ridhaa (moja kwa ajili ya utafiti na nakala moja mpatie mshiriki kama kumbukumbu yake).

**TAARIFA ZA BINAFSI ZA MSHIRIKI**

Muelekeze mlengwa kujaza fomu ya taarifa binafsi na uhakikishe amesaini fomu ya ridhaa na umpe nakala yake.

**Maswali ya utangulizi**

- Unapokuwa hauna kazi hupenda kufanya nini kupumzika?
- Kitu gani kinakufanya uipende kazi yako?

Sasa, tujadili kuhusu uzoefu wako katika kutoa huduma za kliniki wakati wa ujauzito, kujifungua na baada ya kujifungua kwa wasichana wenye umri mdogo;

| **Mada** | **Maswali ya uongozi** |
| --- | --- |
| **Utaratibu wa utoaji wa huduma kwa mama mjamzito na mtoto** | Kwa kuanza majadiliano yetu naomba unielezee utolewaji wa huduma za kliniki wakati wa ujauzito, kujifungua na baada ya kujifungua?  **Dodosa;**   - Je, huduma za cliniki ya mama na mtoto wakati wa ujauzito, kujifungua na baada ya kujifungua ni rafiki kwa wasichana wadogo? - Je huduma hizo zinatolewa kwa mpangilio gani? - Ni kwa siku ngapi ndani ya wiki huduma hizi hutolewa? - Mnafanya kazi kwa masaa mangapi? - Vipi kuhusu siku za mwisho wa wiki? - Ni watumishi wangapi wanaofanya kazi katika idara ya mama na mtoto? |
| ***Uzoefu katika utoaji wa huduma za kliniki wakati wa ujauzito kwa wasichana wadogo****.* | Unaweza kuelezea zaidi juu ya huduma za kliniki wakati wa ujauzito kwa wasichana wadogo wa miaka 15-19??  Dodosa.   - Je, kuna sera au muongozo ambao unasimamia utolewaji wa huduma za kliniki wakati wa ujauzito kwa wasichana wadogo pamoja na watoto wao? - Je wasichana wadogo huja kliniki wakati wa ujauzito? Na huja mapema kiasi gani? - Je unafikiria ni vitu gani huwawezesha wasichana wadogo kuhudhuria kliniki wakati wa ujauzito? - Je unafikiria ni vitu gani vinavyowazuia wasichana wadogo kuhudhuria kliniki wakati wa ujauzito? - Nini mawazo yako juu ya wasichana wadogo kufanya maamuzi ya kuhudhuria kliniki wakati wa ujauzito? Je ni kina nani wanao washawishi? Na wanawashawishi kwa namna gani ? - Je ni changamoto zipi unazo kabiliana nazo wakati wa utoaji wa huduma za kliniki wakati wa ujauzito kwa wasichana wadogo? - Unadhani nini kifanyike kuboresha huduma za afya wakati wa ujauzito kwa wanawake wenye umri mdogo ? |
| *Uzoefu wa huduma za kujifungua kwa wasichana wadogo.* | - Je unafikiria nini kuhusu wanawake wenye umri mdogo kujifungulia nyumbani au kwenye vituo vya kutolea huduma za afya?   Dodosa   - Je wanawake wenye umri mdogo hufika kujifungulia katika vituo vya kutolea huduma za afya? Kama ndiyo, ni mapema kiasi gani ? Kama hapana, ni kwa nini? - Unafikiri ni vitu gani vinavyosaidia wanawake wenye umri mdogo kuja kujifungulia katika vituo vinavyotoa huduma za afya? - Unafikiri ni vitu gani vinavyowazuia wanawake wenye umri mdogo kuja kujifungulia katika vituo vinavyotoa huduma za afya? - Nini mawazo yako juu ya wanawake wenye umri mdogo kufanya maamuzi ya kuja kujifungua katika vituo vinavyotoa huduma za afya? Je ni kina nani wanaowashawishi? Na wanawashawishi vipi? - Unafikiri ni nani anaye washawishi wanawake wenye umri mdogo kujifungulia nyumbani/kituo cha afya? - Ni changamoto gani unakutana nazo katika kutoa huduma za kujifungua kwa wanawake wenye umri mdogo? - Unadhani nini kifanyike kuboresha huduma za afya wakati wa kujifungua kwa wanawake wenye umri mdogo ? |
| ***Uzoefu wa huduma za kliniki kwa wasichana wadogo baada ya kujifungua.*** | Unafikiri nini kuhusu huduma za kliniki kwa wasichana wadogo baada ya kijifungua?  Dodosa,   - Je wasichana wadogo huhudhuria kliniki baada ya kujifungua, kama hapana, unalizungumziaje hilo? - Je wasichana wadogo waliojifungulia nyumbani hufika katika kituo cha afya kwa ajili ya huduma za baada ya kujifungua? Kama ndio, mnawapokeaje? Na mnawashauri nini? - Kutokana na uzoefu wako wa utoaji wa huduma za kliniki kwa wasichana wadogo baada ya kujifungua, ni vitu gani unafikiria huwasaidia kuja kupata huduma za kliniki baada ya kujifungua katika kituo chako? - Ni vitu gani unafikiria vinawazuia wasichana wadogo kuja kupata huduma za kliniki baada ya kujifungua? - Nini mawazo yako juu ya wasichana wadogo kufanya maamuzi ya kuhudhuria kliniki baada ya kujifungua katika vituo vinavyotoa huduma za afya? Je ni kina nani wanaowashawishi? Na wanawashawishi vipi? - Ni changamoto gani unakutana nazo katika kutoa huduma za mama na mtoto kwa wanawake wenye umri mdogo baada ya kujifungua ? - Unadhani nini kifanyike kuboresha huduma za afya za mama na mtoto baada ya kujifungua kwa wanawake wenye umri mdogo ? |
| MAPENDEKEZO | Nini kifanyike ili kuboresha huduma za afya kwa wasichana wa umri mdogo hasa kwenye maeneo haya: |
| ***MWONGOZO KUHUSU MUUNDO*** | (Uliza maswali yako kwa kuzingatia nyanja ya mtu binafsi, familia, jamii na muundo wa huduma za afya)  **Mtu binafsi**: Uelewa kuhusu huduma za klinic kabla wakati na baada ya kujifungua, umuhimu, uhuru/imani kwa mtoa huduma, aibu, uwezo wa kuamua n.k.  **Mahusiano na familia:** Mila na desturi za familia, msaada wa kujifungulia nyumbani, uelewa na imani ya mwenzi, ushawishi wa mama mkwe na watu wengine, umaskini  **Jamii na jumuiya:** imani za kidini, mila na desturi, ushawishi wa majirani, sharia za jamii.  **Taasisi na muundo wa huduma za afya:** upatikanaji wa huduma, huduma rafiki kwa wenye umri mdogo, tabia za mtoa huduma, jinsi ya mhudumu, umbali kutoka kituo cha afya. |
| ***Ufuatiliaji*** | Una mapendekezo gani kuhusu namna ya kuboresha huduma za kliniki wakati wa ujauzito, kujifungua na baada ya ujauzito kwa wajawazito na wazazi wenye umri mdogo? |
| Hitimisho  Unadhani kuna kitu kingine muhimu ambacho hatukuongelea kuhusu vizuizi, viwezeshi na washawishi katika kutafuta huduma za kliniki wakati wa ujauzito, wakati wa kujifungulia kituo cha afya na kliniki baada ya kujifungua?   - Hitimisha - Mshukuru mshiriki - Toa taarifa za mawasiliano na taarifa nyingine muhimu | |

KEY INFORMANT INTERVIEW FOR HEALTH FACILITY STAFF

| Participant IDNO \|__\|__\|__\|__\| Researcher Initials \|__\|__\|__\|  Health facility number \|__\|__\| Date \|__\|__/__\|__/__\|__\|  Type of health facility BEMONC CEMONC  **Introduction**  I am ______________________________ facilitator from CUHAS/Bugando University, Mwanza, working for Mama na Mtoto.  I am _______________________________ from CUHAS/Bugando University, Mwanza, working for Mama na Mtoto. Note-taker for this session.  **Participants introduce themselves by first name.**  **Review consent form with participant. Be sure to cover all the following points:**   - General purpose of the study - Study procedures - Benefits of participation - Risks of participation - Confidentiality - Voluntary participation - Questions   **Obtain two signed consent forms before proceeding. One copy is for the participant and one copy is for the research team.**   - General purpose of the study: This study is looking to explore opinions and perceptions and experiences with pregnant and parenting adolescents in relation to barriers, enablers and influencers for seeking ANC, health facility delivery and PNC services among adolescents here in Misungwi district. - Aims of the interview and expected duration: We are interested in learning about individual, family, community and health system related factors that could either facilitate or hinder adolescents to seek and access ANC, health facility delivery and PNC services. We will also like to know if there are key adolescents’ influencers who would have influence on adolescents’ decision-making process. The interview is expected to take about 1.30hrs. - The interview will be led by one facilitator who will be taking some notes about what you will be talking about and tape recording the discussions. This is for the purpose of helping us remember key issues transpired in the discussions. - Your cooperation is very important and I encourage your active participation throughout our discussion. - There will be no right or wrong answers. All contributions are valued equally. - Information provided will be compiled and before final report transcripts will be shared with you for validation. The final report will be shared with the key implementers of reproductive health services at the district and regional offices, copies will be submitted to the local authorities for future planning of better health services for adolescents. - Any questions? - Consent process   **Warm up Questions**  **Demographic & work history**  Can I ask some details about you and your job?  Job Title ____________________________  Highest Educational Grade attained ___ __ Year of graduation____________  Residence District..........................Ward...........................Village/mtaa....................  Current marital status Single □ Yes □ No  □ Married  □ Divorced/separated  □ cohabiting  Are you originally from this area/district? □ Yes □ No  How old are you? □ (exact age) ……………….  How long have you been working in maternal and child health ……………….  **Now we are going to talk about your experience in providing maternal and child health services to adolescents, by this we mean antenatal care, delivery and postnatal care.** | |
| --- | --- |
|  | |
| **Domain** | **Topic and Probes** |
| Organization of maternal and child health services | To start up our discussion can you explain the provision of antenatal care, delivery and postnatal care **serviices** at your health facility to adolescents.  Probe;   - Is there youth friendly services relating to ANC, Delivery and PNC? - How are they organized? - How many days a week do you provide these services? - How many hours are you working per day? - What about week end days? - How many staff works in maternal and child health unit? |
| ANC experiences with adolescent women | Can you explain more about ANC services provision to adolescent girls of 15-19?  **Probes:**   - Is there any policy/guideline/SOP that guides maternal, new born and child health services to adolescents? - Do adolescent women come for ANC? And how earlier do they attend ANC? - What things do you think enable adolescents to attend ANC? - What things do you think are barriers for them to attend ANC? - What are your thoughts regarding adolescents making decision especially for ANC attendance? Who are key influencers? How do they influence them? - What are the challenges you are facing in provision of ANC to adolescent women? - What do you think can be done to improve health services related to ANC for adolescents? |
| Experience with health facility delivery for adolescents | - What do you think about home delivery and facility delivery for adolescents?   **Probes:**   - Do adolescent women come to deliver at the health facility? If YES, how early? If NO, why is it so? - What things do you think enable them to deliver at the health facility? - What things do you think are barriers for the adolescent women to deliver at the health facility? - What are your thoughts regarding adolescents making decision for health facility delivery? Who are key influencers? How do they influence them? - Who do you think influence adolescents to deliver at home? - What challenges do you encounter in providing delivery services for the adolescent women? - What do you think can be done to improve health services related to delivery for adolescent women? |
| PNC Experience with adolescents | What do you think about post-natal care (PNC) services for adolescent women?   - Do adolescent women come for PNC at this health facility? (If not what can you say about it?) - Do adolescents women who had home delivery come for PNC? If Yes, How do you receive them? What else do you tell them? - According to your experience in providing PNC to adolescent women, what things do you think enable them to come for PNC at your health facility? - What things do you think do prevent them from coming for PNC at your health facility? - What are your thoughts regarding adolescents making decision especially for PNC? Who are key influencers? How do they influence them? - What challenges do you encounter in providing PNC services for the adolescent women? - What do you think can be done to improve health services related to PNC for adolescent women? |
| GUIDE FROM THE MODEL | ***(Guide the discussion to be around individual level, interpersonal and family, community/society and health system level)***  **Individual-** knowledge about PNC, perceived need, comfortability with health care providers, shyness, mistrust, decision autonomy etc.  **Interpersonal and Family level-** family traditions, family support for home delivery, husband’s knowledge and perceptions, influence of mothers in law and other family members and relatives  **Community and social level-** poverty, religious belief, Traditions and cultural practices, influence of neighbors, community by-laws  **Organizational and health system level-** availability of services, youth friendly clinics, behavior of health care providers, sex of health care provider, quality of health services, distance of health facilities |
| Follow up | What would you suggest are the ways to improve adolescent ANC, facility delivery and Post -natal care? |
|  |  |
| **Closing**  Is there anything else you think is important about barriers and enablers for seeking antenatal care, health facility delivery or post-natal care that we have not talked about?   - Summarise - Thank participant - Provide extra information and contacts to participants | |

| **FGD IDNO** \|__\|__\|__\|__\| **Vifupisho vya mtafiti** \|__\|__\|__\|  **Vifupisho vya mwandishi** \|__\|__\|__\|  **Kundi la washiriki: (zungusha):** Baba katika umri mdogo/ Mama/ Baba  **Audio file**: \|__\|__\|__\|  **Namba ya kijiji:**\|__\|__\| **Tarehe**\|__\|__/__\|__/__\|__\|  **Utangulizi**  Mimi_________________(mwezeshaji) kutoka Chuo kikuu cha sayansi na tiba Bugando-Mwanza ninafanya kazi na mradi wa Mama na Mtoto.  Mimi __________________________kutoka__________________(mwandishi)   - Washiriki wajitambulishe kwa majina yao ya kwanza - Chukua taarifa binafsi za washiriki- tumia jina la kwanza kwenye mahojiano   Mimi_________________(mwezeshaji) kutoka Chuo kikuu cha Sayansi na tiba Bugando-Mwanza ninafanya kazi na mradi wa Mama na Mtoto.  Mimi __________________________kutoka__________________(mwandishi)  **Washiriki wajitambulishe kwa majina yao ya kwanzavifuatavyo;**  **Pitia kiapo cha ridhaa na mshiriki na hakikisha mmejadili vipengele**   - **Dhumuni la utafiti** - **Taratibu za utafiti** - **Faida za ushiriki** - **Madhara ya kushiriki** - **Usiri** - **Uhiari wa kushiriki**   Chukua fomu ya ridhaa (moja kwa ajili ya utafiti na nakala moja mpatie mshiriki kama kumbukumbu yake).   - Kuomba wana kikundi kujitambulisha kwa jina la kwanza - Chukua taarifa bimafsi-tumia jina la kwanza wakati wa mahojiano - Wana kikundi watengeneze sheria zao, kwa mfano:   - Kuongea mtu mmoja mmoja kwa wakati   - Ni muhimu kwetu kusikia wazo la kila mmoja na maoni. Hakuna jibu sahihi au lisilo sahihi- mawazo, uzoefu na maoni yako ndio muhimu.   - Ni muhimu kwetu kusikia pande zote –hasi na chanya   - Taarifa zitahifadhiwa kwa usiri. “kile kinachoongelewa hapa kitabaki kuwa siri” - Kuna mwenye swali lolote? - Waeleze washiriki kuhusu kinasa sauti na uwaulize kama wanaridhia kutumika katika majadiliano na ukiwashe na kukikagua kinasa sauti kama kinafanya kazi - Viburudisho /nauli vitatolewa baada ya mahojiano   **Sasa tutaanza majadiliano yetu kuhusu uzoefu na mitamazo yenu kuhusu ujauzito, wakati wa kujifungua na huduma za kliniki baada ya kujifungua.** | |
| --- | --- |
| Viwezeshi, vizuizi na washawishi wa wasichana wadogo na wajawazito kuhudhuria katika vituo vya afya kwa ajili ya huduma za kliniki wakati wa ujauzito. | 1. Je, tunaweza kujadiliana kuhusu utafutaji na upatikanaji wa huduma za afya kwa wasichana wenye ujauzito katika umri mdogo. Dodosa:    - Je, unafikiria wasichana wenye ujauzito katika umri mdogo huenda wapi kutafuta huduma za afya kabla ya kujifungua?    - Je, unaionaje/ unaizungumziaje huduma ya kliniki kwa wasichana wajawazito katika umri mdogo    - Je, unafikiria ni muhimu kwa wasichana wenye ujauzito katika umri mdogo kuhudhuria kliniki kabla ya kujifungua?    - Kama ndiyo, unafikiria ni wakati gani muafaka wasichana wenye umri mdogo wanapaswa kuanza kuhudhuria kliniki kabla ya kujifungua ?    - Je, ni kwa namna gani unamsaidia msichana mwenye ujauzito katika umri mdogo kuhudhuria kliniki kabla ya kujifungua? 2. Je, ni vitu gani humuwezesha msichana mwenye ujauzito katika umri mdogo kuhudhuria kliniki kabla ya kujifungua?   Dodosa:   - Je, unafikiria ni vitu gani vingine vifanyike ilikumuwezesha msichana mwenye ujauzito katika umri mdogo kuhudhuria kliniki kabla ya kujifungua? - Je, umechukua hatua gani kuhakikisha msichana mwenye ujauzito katika umri mdogo anahudhuria kliniki kabla ya kujifungua? - Je, ni vitu gani unafikiria vinamzuia msichana mwenye ujauzito katika umri mdogo kuhudhuria kliniki kabla ya kujifungua? - Je, una fikiria kuna madhara yoyote yatampata msichana mwenye ujauzito katika umri mdogo kama asipohudhuria kliniki kabla ya kujifungua? - Je, ni yapi mapendekezo yako ili kutatua changamoto zinazomzuia msichana mwenye ujauzito katika umri mdogo kuhudhuria kliniki kabla ya kujifungua?  1. Je, ni kwa namna gani maamuzi yanafanyika katika familia kumuwezesha msichana mwenye ujauzito katika umri mdogo kuhudhuria/kutohudhuria kliniki kabla ya kujifungua?  - Je, ni washawishi gani wengine huchangia katika kufanya maamuzi ya wasichana wenye ujauzito katika umri mdogo kuhudhuria kliniki kabla ya kujifungua? - Je, wanashawishi vipi? - Je, ni nini mawazo yako juu ya ushawishi wao? |
| Viwezeshi, vizuizi na washawishi wa wasichana wadogo na wajawazito kuhudhuria katika vituo vya afya wakati wa kujifungua | 1. Je, tunaweza kujadiliana kuhusu upatikanaji wa huduma za afya kwa wasichana wenye ujauzito katika umri mdogo wakati wa kujifungua?    - Je, unapendelea wasichana wenye ujauzito katika umri mdogo kujifungua wakiwa nyumbani au katika vituo vinavyotoa huduma za afya?    - Kama ndio/ hapana dodosa zaidi kwa nini?    - Je, ni nini kinachangia zaidi mapendekezo yako kuhusu mahali pa kujifungua?    - Je, unafikia ni wapi hasa wasichana wenye ujauzito katika umri mdogo wanapenda kujifungua? Kwanini? 2. Je, ni vitu gani vinamuwezesha msichana mwenye ujauzito katika umri mdogo kujifungua katika kituo kinachotoa huduma za afya?  - Je, unafikiria ni vitu gani vingine vifanyike ili kuwezesha msichana mwenye ujauzito katika umri mdogo aweze kujifungua katika kituo kinachotoa huduma za afya? - Je, ni hatua zipi umechukua ili kumuwezesha msichana mwenye ujauzito katika umri mdogo kujifungua katika kituo kinachotoa huduma za afya?  1. Je, ni vitu gani vinachangia kuzuia msichana mwenye ujauzito katika umri mdogo ashindwe kufika kituo cha kutolea huduma za afya kujifungua?  - Unafikiria kuna madhara yoyote kwa msichana mwenye ujauzito katika umri mdogo kutojifungua katika kituo kinachotoa huduma za afya? - Dodosa zaidi ni madhara yapi? - Je, ni yapi mapendekezo yako ili kutatua changamoto zinazomzuia msichana mwenye ujauzito katika umri mdogo kufika katika kituo kinachotoa huduma za afya kujifungua  1. Je, ni kwa namna gani maamuzi yanafanyika katika familia kumuwezesha msichana mwenye ujauzito katika umri mdogo kufika katika kituo kinachotoa huduma za afya wakati wa kujifungua?  - Je, ni washawishi gani wengine huchangia katika kufanya maamuzi ya wasichana wenye ujauzito katika umri mdogo kujifungua katika kituo cha afya? - Je, wanashawishi vipi? - Je, ni nini mawazo yako juu ya ushawishi wao? |
| Viwezeshi, vizuizi na washawishi wa wasichana wadogo na wajawazito kuhudhuria katika vituo vya afya baada ya kujifungua | 1. Sasa, tujadiliana kuhusu upatikanaji wa huduma za afya kwa wasichana aliyejifungua katika umri mdogo baada ya kujifungua?  - Je, unafikiria wasichana wenye watoto katika umri mdogo huenda wapi kutafuta huduma za afya baada ya kujifungua? - Je, unaionaje/ unaizungumziaje huduma ya kliniki kwa msichana aliyejifungua katika umri mdogo baada ya kujifungua? - Je, unafikiria ni muhimu kwa wasichana aliyejifungua katika umri mdogo kuhudhuria kliniki baada ya kujifungua? - Kama ndiyo, unafikiria ni wakati gani muafaka msichana aliyejifungua katika umri mdogo anapaswa kuanza kuhudhuria kliniki baada ya kujifungua ? - Je, ni kwa namna gani unamsaidia msichana aliyejifungua katika umri mdogo kuhudhuria kliniki baada ya kujifungua - Unahisi msichana aliyejifungua katika umri mdogo anafahamu umuhimu wa kwenda kliniki baada ya kujifungua?  1. Je, ni vitu gani humuwezesha msichana aliyejifungua katika umri mdogo kuhudhuria kliniki baada ya kujifungua?   Dodosa:   - Je, unafikiria ni vitu gani vingine vifanyike ilikumuwezesha msichana aliyejifungua katika umri mdogo kuhudhuria kliniki baada ya kujifungua? - Je, umechukua hatua gani kuhakikisha msichana aliyejifungua katika umri mdogo anahudhuria kliniki baada ya kujifungua?  1. Je, ni vitu gani unafikiria vinamzuia msichana aliyejifungua katika umri mdogo kuhudhuria kliniki baada ya kujifungua?  - Unafikiria kitu gani kitatokea kwa msichana aliyejifungua katika umri mdogo kama asipohudhuria kliniki baada ya kujifungua? - Je, ni yapi mapendekezo yako ili kutatua changamoto zinazomzuia msichana mwenye aliyejifungua katika umri mdogo kuhudhuria kliniki baada ya kujifungua?  1. Je, ni kwa namna gani maamuzi yanafanyika katika familia kumuwezesha msichana aliyejifungua katika umri mdogo kuhudhuria kliniki baada ya kujifungua?  - Je, ni washawishi gani wengine huchangia katika kufanya maamuzi ya msichana aliyejifungua katika umri mdogo kuhudhuria kliniki baada ya kujifungua?   - Je, wanashawishi vipi?   - Je, ni nini mawazo yako juu ya ushawishi wao? |
| MAPENDEKEZO | 1. Nini kifanyike ili kuboresha huduma za afya kwa wasichana wa umri mdogo hasa kwenye maeneo haya:  - Kliniki ya wajawazito: - Huduma za kujifungua kituo cha afya - Kliniki baada ya kujifungua |
| MWONGOZO KUHUSU MUUNDO | (Uliza maswali yako kwa kuzingatia nyanja ya mtu binafsi, familia, jamii na muundo wa huduma za afya)  **Mtu binafsi**: Uelewa kuhusu huduma za klinic kabla wakati na baada ya kujifungua, umuhimu, uhuru/imani kwa mtoa huduma, aibu, uwezo wa kuamua n.k.  **Mahusiano na familia**: Mila na desturi za familia, msaada wa kujifungulia nyumbani, uelewa na imani ya mwenzi, ushawishi wa mama mkwe na watu wengine, umaskini  **Jamii na jumuiya**: imani za kidini, mila na desturi, ushawishi wa majirani, sharia za jamii.  **Taasisi na muundo wa huduma za afya**: upatikanaji wa huduma, huduma rafiki kwa wenye umri mdogo, tabia za mtoa huduma, jinsi ya mhudumu, umbali kutoka kituo cha afya |
| **Hitimisho**  Je, unadhani kuna kitu kingine muhimu unachopenda kuongeza, kujadili au kupendekeza ili kuboresha utolewaji wa huduma za kliniki kwa wasichana wenye ujauzito katika umri mdogo wakati wa ujauzito, wakati wa kujifungua na baada ya kuifungua.  ***Asante kwa muda wako na kwa ushiriki wako katika majadiliano haya.*** | |

| **FGD IDNO** \|__\|__\|__\|__\| **Facilitator Initials** \|__\|__\|__\|  **Note-taker Initials**\|__\|__\|__\|  **Participant group: (circle):** Young husbands/Elder women/Elder men  **Audio file**: \|__\|__\|__\|  **Village:** _________________ **Date**\|__\|__/__\|__/__\|__\|  **Introduction**  I am ______________________________ from Mama na Mtoto (Facilitator)  I am ______________________________ from Mama na Mtoto (note-taker)  **Participants Introduce themselves and are given numbers**  **Review consent forms with participants. Be sure to cover all the following points:**   - General purpose of the study - Study procedures - Benefits of participation - Risks of participation - Confidentiality - Voluntary participation - Questions   **Obtain signed consent.**  **Ask group to define their own ground rules, for example:**   - Only one person talks at a time - It is important for us to hear everyone’s ideas and opinions. There are no right or wrong answers to questions – just ideas, experiences and opinions, which are all valuable. - It is important for us to hear all sides of an issue – the positive and negative. - Confidentiality is assured. What is shared here stays here.   **Ask if any person has any questions.**  **Explain tape recording procedure, ensure all participants are comfortable being recorded. Turn on recorder and check audio levels.**  **Refreshments will be served after the discussion.**  **Now we are going to start our discussion concerning your experience and perspectives on adolescent pregnant women, including accessing ANC, health facility deliveries, and PNC.**  Participant should fill the demographic sheet with  Warm up questions   - What is your favourite place to go? - What types of crops grow at this village? - What is the name of your best friend? | |
| --- | --- |
| Enablers, barriers, and influencers of adolescent women to attend for ANC | - Can you tell us about how pregnant adolescents seek and access ANC services? Probes:   - What do you think of adolescents going for ANC during pregnancy?   - What do you think about ANC for pregnant adolescents?   - Do you think it is important for pregnant adolescents to attend ANC?   - If yes: When do you think they should go for their first appointment?   - In what ways are you helping pregnant adolescents to attend ANC? - Are there things that help pregnant adolescents before delivery to attend ANC? Probes:   - Do you think there are other things that will help pregnant adolescents to attend ANC?   - Did you take any efforts to ensure adolescent pregnant women in your life attend ANC?   - Do you think there are things that hinder adolescent pregnant women from attending ANC?   - What do you think are the problems that result from not attending ANC for pregnant adolescents?   - What do you think would help to address the things that stop adolescent girls from attending ANC? - How do families come to the decision that pregnant adolescent women should or should not attend ANC? Probes:   - Who are other people that influence adolescent girls’ decisions to attend ANC?   - How do they influence the decision?   - What are your thoughts on the influence of these people? |
| Enablers, barriers, and influencers of adolescent women for health facility delivery | - Can we discuss availability of health facility delivery services for pregnant adolescents? Probes:   - For pregnant adolescents, what place of delivery is preferred? Home? Health facility? Other? Why?   - What influences your preference concerning place of delivery for pregnant adolescents?   - What place of delivery do you think pregnant adolescents prefer? Why? - What do you think are the things that will help adolescent pregnant women to deliver at health facilities? Probes:   - Are there things that could be done to enable adolescent pregnant girls to deliver at health facilities?   - What efforts have you made to help adolescent girls deliver at health facilities? - What do you think are the things that stop adolescent girls from delivering at health facilities? Probes:   - What do you think are the things that result from pregnant adolescents delivering at home? Anything else?   - What do you recommend we do to address the barriers that stop adolescent women from delivering at health facilities? - How do families come to the decision that adolescent girls should or should not deliver in health facilities? Probes:   - Who are other people that influence the decision of place of delivery for adolescent pregnant women?   - How do they influence the decision?   - What do you think about their influence? |
| Enablers, barriers, and influencers of adolescent women for attending PNC | - Now let’s talk about PNC services for adolescent mothers. Probes:   - Where do adolescent mothers go for health services after delivery?   - What do you think about PNC services for adolescent mothers?   - Do you think it is important for adolescent mothers to attend PNC? Why?   - If yes, when do you think adolescent mothers should attend PNC?   - In what ways do you help adolescent mothers to attend PNC?   - Do you think adolescent mothers know the importance of attending PNC? - What could help adolescent mothers to attend PNC? Probes:   - What are the things that can be done to help adolescent mothers attend PNC?   - What efforts have you made to ensure adolescent mothers attend PNC? - What are the things that stop adolescent mothers from attending PNC?   - What do you think happens as a result of adolescent mothers not attending PNC?   - What actions do you recommend to address the things that stop adolescent mothers from attending PNC? - How do families come to the decision for adolescent mothers to attend or not attend PNC?   - Who are other people that influence the decision of adolescent mothers to attend PNC?   - In what ways do they influence the decision?   - What do you think about this influence? |
| **Closing**  Is there anything else you can recommend to improve ANC, health facility delivery, or PNC services for adolescent pregnant and parenting women.  ***Thank you for your time and your participation in this discussion.*** | |
